# Supplementary material for: Bemcentinib as monotherapy and in combination with low-dose cytarabine in acute myeloid leukemia patients unfit for intensive chemotherapy: a phase 1b/2a trial
Source: Nat Commun. 2025 Mar 23;16:2846. doi: 10.1038/s41467-025-58179-6 (PMC11930985; doi:10.1038/s41467-025-58179-6)
Supplement: Supplementary file 1 — Supplementary Information [file 41467_2025_58179_MOESM1_ESM.pdf]

Supplementary Information

Contents

Supplementary Figures 2
Supplementary Figure 1. Dose response curves of maximal inhibition in Undefined/Immature Myeloid cells. 2
Supplementary Tables 3
Supplementary Table 1: Study Centers and associated ethical committees/institutional review boards. 3
Supplementary Table 2: Key changes to protocol 5
Supplementary Table 3: Inclusion/Exclusion criteria 8
Supplementary Table 4a: Response criteria in AML 9
Supplementary Table 4b: Response criteria in MDS 10
Supplementary Table 5 Antibodies used for CyTOF analysis. 12
Supplementary References 13
Supplementary Notes 14
Supplementary Note 1: Norwegian Protocol Version 1.1 14

Supplementary Figures

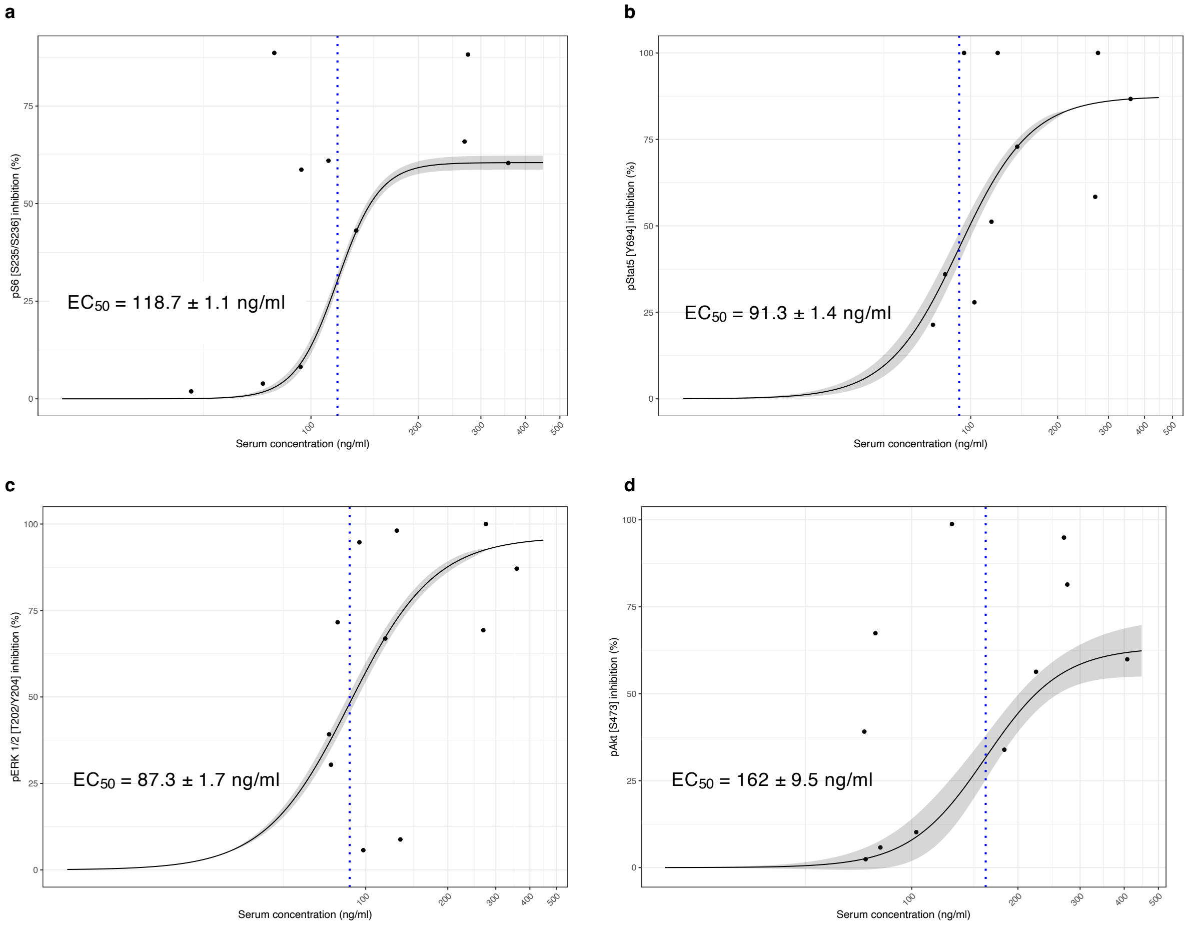

Supplementary Figure 1. Dose response curves of maximal inhibition in Undefined/Immature Myeloid cells.

Dose response curves are shown for downstream markers of AXL signalling pS6 S235/S236 (a), pSTAT5 Y694 (b), pERK1/2 T202/Y204 (c) and pAKT S473 (d) using a robust log-logistic dose response model with lower limit set to 0. The calculated EC_{50} (± standard error) is indicated (text and dotted line). The black line indicates the fitted model with the 95% confidence-interval indicated by grey shading. N = 12 individual patients. Patients for whom maximum inhibition occurred before treatment onset are not shown because the corresponding dose (0 ng/ml) cannot be accommodated on the logarithmic dose scale. However, they were included for calculation of the dose-response curves. Source data are provided as a Source Data file.

Supplementary Tables

**Study Center**	**Ethical approval**
University of Iowa Hospitals and Clinics, Iowa City, USA	Human Subjects Office / Institutional Review Board<br>Hardin Library, Suite 105A<br>600 Newton Rd<br>Iowa City, IA 52242-1098, USA
The University of Texas M.D. Anderson Cancer Center, Houston, USA	The University of Texas, MD Anderson Cancer Center, Institutional Review Board<br>7007 Bertner Ave., Unit 1637<br>Houston, TX 77030, USA
University Hospital Frankfurt, Germany	Ethik-Kommission des Fachbereichs Medizin der Goethe-Universität Frankfurt am Main<br>Theodor-Stern-Kai 7<br>60590 Frankfurt am Main, Germany
Hannover Medical School, Germany	Ethik-Kommission der Medizinischen Hochschule Hannover<br>Carl-Neuberg-Str. 1<br>30625 Hannover, Germany
University Hospital Mannheim, Germany	Ethik-Kommission II der Universität Heidelberg Medizinische Fakultät Mannheim<br>Haus 42, Ebene 3<br>Theodor-Kutzer-Ufer 1-3<br>68167 Mannheim, Germany
University Hospital of Ulm, Germany	Ethikkommission der Universität Ulm<br>Helmholtzstraße 20 (Oberer Eselsberg)<br>D - 89081 Ulm, Germany
Azienda Sanitaria Ospedaliera Santa Croce e Carle, Cuneo, Italy	Comitato Etico Interaziendale<br>Azienda Ospedaliera S. Croce e Carle<br>via M. Zovetto, 18<br>12100 Cuneo, Italy
San Martino Hospital, University of Genoa, Italy	Comitato Etico Regione Liguria<br>IRCCS Policlinico San Martino,<br>Sistema Sanitario Regione Liguria<br>Largo Rosanna Benzi, 10<br>16132 Genova, Italy
Vito Facci Hospital, Lecce, Italy	Comitato Etico ASL 3 Lecce<br>Via Miglietta, 5<br>73100 Lecce, Italy
University Hospital Parma, Italy	Comitato Etico dell'Area Vasta Emilia Nord (AVEN)<br>Azienda Ospedaliero-Universitaria di Parma<br>Via Gramsci 14<br>43126 Parma, Italy
Haukeland University Hospital, Bergen, Norway.	Regionale Komiteer for Medisinsk og Helsefaglig Forskningsetikk – REK Sør-Øst D<br>Gullhaugveien 1-3<br>0484 Oslo, Norway

Supplementary Table 1: Study Centers and associated ethical committees/institutional review boards.

Amendment #	Date of Amendment	Summary of Key Changes
Version 1.1	26 Jun 2014	-
Version 2.0	14 Dec 2016	• Allowed Study BGBC003 to progress with a formulated product of bemcentinib.<br>• Permitted exploration of a 2 or 3-day loading dose during Part A of the study, to determine the recommended dose for Part B.<br>• The requirement for subjects in Norway to demonstrate bemcentinib sensitivity ex vivo (AML subjects) or to express AXL (MDS subjects), to be eligible for the study, was removed.<br>• The study exclusion criteria were developed further and consolidated across the bemcentinib clinical development program.<br>• Minor adjustments were made for consistency across the cardiac exclusion criteria and the use of antacids prior to first dose.<br>• The DLT criteria was revised to harmonize toxicity categories across all countries participating in the study, whilst ensuring the requirements of each regulatory authority were met.<br>• Blood volumes for PD testing were clarified. The protocol now only required mandatory bone marrow assessment at Screening and Cycle 2 Day 1. Predose bone marrow assessments remained at the start of every cycle thereafter and was to be conducted as clinically indicated.<br>• Other changes were made to improve the quality of the document and to reflect the latest safety information and guidance provided in the current Investigator Brochure of bemcentinib.
Version 3.0	23 May 2017	• Clarification was provided for the previously approved bemcentinib loading dose scheme for Formulation 2.<br>• The subject fasting requirements around dosing were added.<br>• Details regarding the management of subjects who require the support of corticosteroids at above 10 mg daily were added.
Version 4.0	29 Aug 2017	• Current study database was based on US protocol that contaieds 4 groups in Part B, while the Norwegian protocol v3.0 had only 3 groups in Part B. This protocol amendment was prepared to re-name groups in Part B in order to enable the site to enter data into study database for subjects enrolling into Part B.
Version 5.0	06 Dec 2017	• Protocol title was updated to reflect inclusion of decitabine.<br>• The cohort for Part B3 (bemcentinib + decitabine) was added.<br>• Cytarabine PK samples were removed from subjects in Part B2.<br>• The inclusion criteria for Part B1, B2 and B4 were revised.<br>• Guidance on administration and dosing of cytarabine/decitabine was included.
Version 6.0	18 Sep 2019	• An additional Part B5 cohort was added for further exploring the safety and preliminary efficacy of subjects treated in second (or later) line with combination of bemcentinib and low dose cytarabine.

		• Recruitment of 28 subjects into the new Cohort B5 to account for the heterogeneity in the study population seen in Part B2 (ie,14 subjects).
Version 7.0	23 Mar 2020	• A new efficacy endpoint of event free survival was added (replacing original progression-free survival).<br>• The sample size was amended in Part B5 from 28 subjects to 20 subjects and overall number of evaluable subjects from 120 to 102 subjects.<br>• Definition of refractory AML subjects was added.<br>• The number of refractory subjects and/or subjects who had received 2 or more prior treatments for AML in Part B5 was restricted to 1/3rd of the sample size (ie, no more than 6 evaluable subjects).<br>• Cytarabine dosing in Part B5 was changed to 20 mg subcutaneously twice daily for 10 days every 28 days.<br>• The number of cycles of study treatment to be administered was clarified.<br>• Additional collection points for bone marrow aspirates and response assessments were added.
Version 8.0	04 Aug 2020	• Clarity was added on the rationale for allowing subjects experiencing disease progression to be considered for continuation of treatment with bemcentinib (note: this could only occur where the investigator believed subjects could continue to derive some clinical benefit from continued bemcentinib treatment and where the decision had been agreed between the investigator, sponsor and subject).<br>• Confirmation was included that subjects experiencing disease progression who continued bemcentinib treatment would be evaluated for safety and efficacy in the same manner as other BGBC003 subjects.<br>• Added a description of how data from subjects who continued treatment beyond disease progression would be handled statistically for both efficacy and safety (ie, the final analysis would include all subjects, but a separate output would be provided for those subjects who continued treatment beyond disease progression).
Version 9.0	14 Jul 2021	• References to Chiltern/Covance as the CRO responsible for SAE management were replaced with the Syneos Health.

Supplementary Table 2: Key changes to protocol

Changes compared to the pre-registered Norwegian protocol (Supplementary Note 1)

**Inclusion criteria [1]**	
**1.**	**Provision of signed written informed consent.**
**2.**	**Histological, molecular or cytological confirmation of :**
**Cohort A (dose escalation cohort)**	• Patients with AML or MDS who must have received previous treatment with cytotoxic chemotherapy (with or without hematopoietic stem cell transplantation) or a gene expression modulator, such as a demethylating agent.<br>• Subjects suitable for intensive chemotherapy were to be in second or subsequent relapse or be refractory to at least 2 induction regimens.<br>• If eligible, they were to have undergone hematopoietic stem cell transplantation.<br>• Subjects receiving an allograft in first remission were eligible at the time of relapse.<br>• Subjects who were unsuitable for intensive chemotherapy as a result of advanced age or co-morbidities were to have relapsed following at least 1 line of therapy or be refractory.
**Cohort B2 (LDAC cohort)**	• Subjects with AML who were unsuitable for intensive chemotherapy as a result of advancing age or co-morbidities and who were suitable to receive treatment with cytarabine
**Cohort B5 (LDAC cohort)**	• Subjects with relapsed or refractory AML who were unsuitable for intensive chemotherapy as a result of advanced age or co-morbidities meeting the following criteria:<br>o Must have received at least one prior treatment for AML<br>o Were suitable to receive treatment with "low dose" cytarabine (LDAC). LDAC was defined as 20 mg cytarabine administered subcutaneously twice daily for 10 days every 28 days.<br>o The number of subjects with refractory AML, defined as no hematological response to last AML treatment and/or subjects who had received 2 or more prior treatments for AML, were restricted to 1/3 of the sample size (no more than 6 evaluable subjects).
**3.**	**Eastern Cooperative Oncology Group (ECOG) performance status 0, 1 or 2.**
**4.**	**Age 18 years or older.**
**5.**	**Reproductive potential**<br>• Female subjects of childbearing potential were required to have a negative serum pregnancy test within 3 days prior to taking their first dose of bemcentinib.<br>• Male subjects and female subjects of reproductive potential were expected to agree to practice highly effective methods of contraception (such as hormonal implants, combined oral contraceptives, injectable contraceptives, intrauterine device with hormone spirals, total sexual abstinence, vasectomy) throughout the study and for >3 months after the last dose of bemcentinib.<br>• Female subjects were considered NOT of childbearing potential if they had a history of surgical sterility or evidence of post-menopausal status defined as any of the following:<br>o Natural menopause with last menses >1 year ago.<br>o Radiation induced oophorectomy with last menses >1 year ago.<br>o Chemotherapy-induced menopause with last menses >1 year ago.
**Exclusion Criteria**	
**1.**	**Subjects who had a matched donor and who were candidates for allogenic BM transplantation.**
**2.**	**Pregnant or lactating.**
**3.**	**History of the following cardiac conditions:**

	• Congestive cardiac failure of >Class II severity according to the New York Heart Association (NYHA).<br>• Ischemic cardiac event including myocardial infarction within 3 months prior to first dose. Subjects with prior history or ECG evidence of old myocardial infarction were to be discussed with the sponsor to confirm eligibility.<br>• Uncontrolled cardiac disease, including unstable angina, uncontrolled hypertension (sustained systolic BP >160 mmHg or diastolic BP >90 mmHg), or need to change medication within 6 weeks of provision of consent due to lack of BP control.<br>• History or presence of sustained bradycardia (≤55 beats per minute), left bundle branch block, cardiac pacemaker, or ventricular arrhythmia. Note: Subjects with supraventricular arrhythmia needed to seek confirmation from sponsor for eligibility.<br>• Family history of long QTc syndrome; personal history of long QTc syndrome or previous drug induced QTc prolongation of at least grade 3 (QTc >500 ms).<br>• Presence of any factors that increase the risk for QTc prolongation such as resistant or inadequately treated heart failure, presence of hypokalemia or hypomagnesemia not corrected by, or not responding to, replacement therapy or inadequately treated hypothyroidism (thyroid-stimulating hormone not within the expected range of the institution).
**4.**	Abnormal left ventricular ejection fraction on echocardiography (less than the lower limit of normal for a subject of that age at the treating institution or <45%, whichever was lower).
**5.**	Current treatment with any agent that was known to cause QT prolongation and had a risk for Torsade de Pointes that could not be discontinued at least 5 half-lives or 2 weeks prior to the first dose of study treatment.
**6.**	Screening 12-lead ECG with a measurable QTcF >450 ms.
**7.**	Ongoing infection requiring systemic treatment. Subjects who were on prophylactic antimicrobials or who had been afebrile for 48 hours following the initiation of antimicrobials were eligible.
**8.**	Inadequate liver function as demonstrated by serum bilirubin ≥1.5 times the upper limits of normal range (ULN) or alanine aminotransferase (ALT) or aspartate aminotransferase (AST) ≥2.5 times the ULN (or ≥5 times the ULN for AST or ALT in the presence of liver involvement by leukemia).
**9.**	Inability to tolerate oral medication.
**10.**	Existing gastrointestinal disease at that time that affected drug absorption such as celiac disease or Crohn's disease.
**11.**	Known lactose intolerance.
**12.**	Required vitamin K antagonists. Note: Subjects receiving low doses prescribed to maintain the patency of venous access devices could be included.
**13.**	Treatment with any of the following: histamine receptor 2 (H_2) antagonist, proton pump inhibitors or antacids within 3 days of administration.
**14.**	Treatment with any medication which was predominantly metabolized by CYP3A4 and had a narrow therapeutic index.
**15.**	Previous bowel resection that would interfere with drug absorption.
**16.**	Evidence of ongoing gastrointestinal graft versus host disease.
**17.**	Hematopoietic stem cell transplantation within 6 months.
**18.**	Impaired renal function as demonstrated by a creatinine clearance of <30 mL/min determined by Cockcroft-Gault formula.
**19.**	Radiotherapy or chemotherapy within the 14 days prior to the first dose of bemcentinib being administered (other than hydroxyurea).
**20.**	Was receiving an investigational anticancer treatment concurrently or within 14 days or 5 half-lives (whichever was shorter) of either the parent drug or any known active metabolite prior to the start of bemcentinib.
**21.**	Unresolved CTCAE >grade 2 toxicity (other than stable toxicity) from previous anticancer therapy excluding alopecia.

**22.**	Any evidence of severe or uncontrolled systemic conditions (e.g., severe hepatic impairment) or current unstable or uncompensated respiratory or cardiac conditions that did not favor subject's participation in the study or could jeopardize compliance with the protocol.
**23.**	Active, uncontrolled CNS disease including CNS leukemia.
**24.**	Active infection with human immunodeficiency virus (HIV), hepatitis B or C viruses – screening for viral infections was not required for entry to this study.
**25.**	Major surgery within 28 days prior to the start of bemcentinib – excluding skin biopsies and procedures for insertion of central venous access devices.
**26.**	Hypersensitivity to cytarabine, decitabine or any of its excipients.
**27.**	Prior exposure to Astellas ASP2215 (FLT3/AXL inhibitor Gilteritinib).

Supplementary Table 3: Inclusion/Exclusion criteria

[1] Subjects who met the inclusion criteria as stated in the relevant country-wise protocol versions were included in the study. The inclusion criteria listed are from Protocol Version 11.0 (Germany).

[2] Subjects who met any of the exclusion criteria stated in the relevant country-wise protocol versions were excluded from the study. The exclusion criteria listed are from Protocol Version 11.0 (Germany).

**Response Criteria in AML**		**Bone Marrow Blasts/Myeloblast**	**Circulating or Peripheral Blood Blasts**[a]	**Absolute Neutrophil Count**	**Platelet Count**	**EMD**
Morphologic Complete Remission	CR	<5% and no myeloblasts with Auer rods	None	≥1.0 x 10^9/L or ≥1,000/µL	≥100 x 10^9/L or ≥100,000/µL	None
CR with Incomplete Blood Count Recovery	CRi	<5% and no myeloblasts with Auer rods	None	<1.0 x 10^9/L or <1,000/µL	<100 x 10^9/L or <100,000/µL	None
Morphologic leukemia-free state	MLFS	<5% and no myeloblasts with Auer rods	No hematologic recovery required			None
Partial Remission	PR	Decrease of myeloblasts % to 5% to 25% and decrease of pretreatment myeloblast % by at least 50%	All hematologic criteria of CR			
Unchanged	UN	Absence of CR, CRi or PR and criteria for PD not met				
Disease Progression[b]	PD	>50% increase in myeloblasts over the pretreatment value (a minimum 15% increase is required if <30% myeloblasts pretreatment)	>50% increase in PB to >25 × 10^9/L or >25 000/µL[c]			New EMD disease

a. Peripheral blasts are quantified as an automated WBC times percentage of blasts by manual differential (100 cells) per standard clinical laboratory procedures.
b. PD is usually accompanied by a decline in ANC and platelets, increased transfusion requirement and decline in performance status and increase in symptoms.
c. Certain targeted therapies may cause a differentiation syndrome, that is, a transient increase in the percentage of bone marrow blasts (myeloblasts) and an absolute increase in PBs; in the setting of therapy with such compounds, an increase in blasts may not necessarily indicate PD.

Supplementary Table 4a: Response criteria in AML

Response Criteria in MDS		Bone Marrow[a]	Peripheral Blood[a]	Additional Considerations
Complete Remission	CR	≤5% myeloblasts with normal maturation of all cell lines with no evidence for dysplasia[b].	Normalization of blood counts as defined by:<br>• Hb ≥11g/dL (not supported by EPO or transfusions)<br>• Platelets ≥100 x 10^9/L or 100,000/µL (not supported by transfusions)<br>• Neutrophils ≥1.5 x 10^9/L or 1,500/µL (not supported by G-CSF)<br>• PB 0%	Responses to be confirmed in two successive assessments; response parameters in peripheral blood needs to be maintained for at least 6 weeks.
Partial Remission	PR	All CR criteria met if abnormal before treatment except for:<br>• Myeloblasts decrease by 50% or more over pretreatment value but still >5%, or<br>• Less advanced FAB or IPPS category compared to pretreatment value	Hematologic improvement	Absolute values must last at least 6 weeks.
Marrow CR	MR	≤5% myeloblasts and decrease by ≥50% over pretreatment value	If hematologic improved, these should be noted in addition to marrow CR.	
Stable Disease	SD	Failure to achieve at least PR, but no evidence of PD for at least 3 treatment cycles.		
Disease Progression	PD	• For patients with less than 5% myeloblasts: a 50% or more increase in myeloblasts to more than 5% myeloblasts.<br>• For patients with 5% to 10% myeloblasts: a 50% or more increase to more than 10% myeloblasts.<br>• For patients with 10% to 20% myeloblasts: a 50% or more increase to more than 20% myeloblasts.<br>• For patients with 20% to 30% myeloblasts: a 50% or more increase to more than 30% myeloblasts.	Myeloblast increase plus at least one of the following:<br>• ≥50% decrease in ANC or platelets<br>• Reduction in Hb by ≥2g/dL<br>• Transfusion dependence.	Death during treatment
Relapse after CR or PR		Relapse is characterized by at least one of the following:<br>• Return to pretreatment myeloblasts percentage<br>• ≥50% decrease in ANC or platelets<br>Reduction in Hb by ≥2g/dL or transfusion dependence.		
Disease transformation		Transformation to AML (30% or more blasts).		

a. Improvements in BM and PB must be based on ≥2 successive assessments; response in PB must be maintained for at least 6 weeks.

Supplementary Table 4b: Response criteria in MDS

Isotope		Antigen	Clone	Loc.	Vendor	Catalog#	RRIB	Working dilution	Marker of:
**Population markers**									
Y	89	CD45	HI30	S	Standard BioTools	3089003B	AB_2938863	1:4000	Pan leukocytes
Er	168	CD34	581	S	BioLegend	343531	AB_2562837	[1]	Hematopoietic stem/progenitor cells
Dy	163	CD33	WM53	S	Standard BioTools	3163023B	AB_3661863	1:800	Myeloid cells
Yb	173	CD300e	233810	S	Thermo Fisher	MA5-23940	AB-2607573		Myeloid lineage
Sm	147	CD20	2H7	S	Standard BioTools	3147001B	AB_2921324	1:400	B cells
Er	170	CD3	UCHT1	S	Standard BioTools	3170001B	AB_2811085	1:3200	T cells
Nd	145	CD4	RPA-T4	S	Standard BioTools	3145001B	AB_3661845	1:800	Helper T cells
Dy	162	CD8a	RPA-T8	S	Standard BioTools	3162015C	AB_2811089	1:3200	Cytotoxic T cells
Gd	155	CD56	B159	S	Standard BioTools	3155008B	AB_2861412	1:400	NK, γδ-T cells, activated CD8+ T cells, Dendritic cells
Tm	169	CD25	2A3	S	Standard BioTools	3169003C	AB_2938861	1:3200	Basophils, Regulatory T cells, and activated helper T cells
Pr	141	CD66b	G10F5	S	BioLegend	305102	AB_314494	[1]	Neutrophils
Nd	143	CD117	104D2	S	Standard BioTools	3143001C	AB_2847864	1:1600	Hematopoietic stem/ multipotent progenitor cell/Common myeloid progenitor cells
Nd	146	CD64	10.1	S	Standard BioTools	3146006C	AB_2661790	1:400	Macrophages/Monocytes
Gd	160	CD14	M5E2	S	BioLegend	301843	AB_2562813	[1]	Monocytes
Nd	148	CD16	3G8	S	Standard BioTools	3148004B	AB_3665424	1:200	Neutrophils and subsets of NK and monocytes
Eu	151	CD123	6H6	S	Standard BioTools	3151001B	AB_3661860	1:800	Basophils, myeloid dendritic cells and plasmacytoid dendritic cells
Bi	209	CD11b	ICRF44	S	Standard BioTools	3209003B	AB_2687654	1:1600	Granulocytes, monocytes, NK cells and dendritic cells
Tb	159	CD90	5E10	S	Standard BioTools	3159007C	AB_2893063	1:400	Thymocytes, hematopoetic stem cells, NK cells
Yb	174	HLA-DR	L243	S	Standard BioTools	3174001C	AB_2665397	1:800	Activation of dendritic cells, monocytes and B cells
Nd	144	CD38	HIT2	S	Standard BioTools	3144014C	AB_2687640	1:800	Activation
**Signaling Markers**									
Sm	149	p4E-BP1 [T37/T46]	236B4	I	Standard BioTools	3149005C	AB_2847866	1:400	mTOR signaling
Eu	153	pSTAT1 [Y701]	58D6	I	Standard BioTools	3153003C	AB_2811248	1:400	JAK/STAT signaling
Gd	158	pSTAT3 [Y705]	4/P-STAT3	I	Standard BioTools	3158005C	AB_2811100	1:400	JAK/STAT signaling
Nd	150	pSTAT5 [Y694]	47	I	Standard BioTools	3150005A	AB_2744690	1:400	JAK/STAT signaling
Sm	152	pAkt [S473]	D9E	I	Standard BioTools	3152005C	AB_2811246	1:200	AKT signaling
Er	166	pNFkBp65 [S529]	K10-895.12.50	I	Standard BioTools	3166006A	AB_2847867	1:400	NFkB/IkB signaling
Dy	164	IkBa	L35A5	I	Cell Signaling Technology	15595	AB_390781		NFkB/IkB signaling
Ho	165	pCREB [S133]	87G3	I	Standard BioTools	3165009C	AB_2661832	1:200	CREB signaling

Isotope	Antigen	Clone	Loc.	Vendor	Catalog#	RRIB	Working dilution	Marker of:
Er 167	pERK1/2 [T202/Y204]	D13.14.4E	I	Standard BioTools	3167005C	AB_2661834	1:400	MAP kinase signaling
Gd 156	pP38 [T180/Y182]	D3F9	I	Standard BioTools	3156002C	AB_2661826	1:1600	MAP kinase signaling
Yb 172	pS6 [S235/S236]	N7-548	I	Standard BioTools	3172008C	AB_2938622	1:400	MAP kinase signaling
Yb 171	Axl	1H12	S	BerGenBio AS	1H12	[2]	[1]	AXL signaling
Dy 161	pAXL [Y779]	AF2228	I	R&D Systems	AF2228	AB_2062560	[1]	AXL signaling
Yb 176	pPLCg1 [Y783]	12F4.2	I	Millipore	MABS63	AB_11214201	[1]	PIP3 signaling
Lu 175	pHistone3 [S28]	HTA28	I	Standard BioTools	3175012A	AB_2847869	1 : 200	Proliferation
Nd 142	Cleaved Caspase 3	D3E9	I	Standard BioTools	3142004A	AB_2847863	1 : 400	Apoptosis
Sm 154	SLFN11	E-4	I	Santa Cruz	Sc-374339	AB_10989536	[1]	Other

Supplementary Table 5 Antibodies used for CyTOF analysis.

Development, validation and testing of the panel are detailed in Tislevoll et al.[1]

Loc., Location; S, Surface; I, Intracellular; RRID, Research Resource Identifier.

[1] These antibodies were conjugated to the isotopes in-house, using the Maxpar X8 antibody conjugation Kit (Standard BioTools). Working dilution must be determined empirically for each batch.

[2] As described in Ahmed et al[2]

Supplementary References

1. Tislevoll, B.S. et al. Early response evaluation by single cell signaling profiling in acute myeloid leukemia. *Nat. Commun.* **14,** 115 (2023).
2. Ahmed, L. et al. Novel anti-human Axl monoclonal antibodies for improved patient biomarker studies. Diagnostic Pathology, [S.l.], v. 2, n. 1,. ISSN 2364-4893. https://doi.org/10.17629/www.diagnosticpathology.eu-2016-2:104.

Supplementary Notes

Supplementary Note 1: Norwegian Protocol Version 1.1

The protocol in force at the time of enrolment of the first patient. See Supplementary Table 1 for a log of significant changes in subsequent versions.

BerGenBio AS
Jonas Lies vie 91
5021 Bergen, Norway

CLINICAL STUDY PROTOCOL

**Investigational Product:**	BGB324
**Protocol Number:**	BGBC003
**Phase**	Phase I
**Protocol Title:**	A Phase I multicenter open-label study of BGB324 as a single agent and in combination with cytarabine in patients with acute myeloid leukemia or high/intermediate (int-2) risk myelodysplastic syndrome which overexpresses axl
**EudraCT Number:**	2014-000165-46
**Version and Date:**	1.1<br>26 June 2014

CONFIDENTIALITY STATEMENT:

Information in this protocol is confidential and should not be disclosed, other than to those directly involved in the execution or the ethical/regulatory review of the study, without written authorisation from BerGenBio AS

This study will be conducted in compliance with International Conference on Harmonisation (ICH) guidelines on Good Clinical Practice (GCP), the Declaration of Helsinki (with amendments), and in accordance with local legal and regulatory requirements

1. Protocol Approval Signatures

Protocol BGBC003 Version 1.1, 26 June 2014

Sponsor's Approval:

This protocol has been approved by BerGenBio AS

Signature: **Date:**

Murray Yule MD, PhD
Chief Medical Officer

Investigator's Approval:

I, the undersigned, have reviewed the Protocol, including Appendices and I will conduct the BGBC003 clinical study as described and in accordance with International Conference on Harmonisation (ICH) guidelines on Good Clinical Practice (GCP) and all the ethical and regulatory considerations stated. I have read and understood the contents of the BGB324 Investigator's Brochure

Signature: **Date:**

2. Study Personnel

**Sponsor's Representative:**	Murray Yule, MD PhD<br>Telephone: +44 7557 818330<br>Email: murray.yule@bergenbio.com
**Sponsor's Main Contact:**	Lynn Wilson, PhD<br>Telephone: +44 7906 812131<br>Email: lynn.wilson@bergenbio.com
**Sponsor's Medical Contact:**	Murray Yule, MD PhD<br>Telephone: +44 7557 818330<br>Email: murray.yule@bergenbio.com
**Sponsor site:**	BerGenBio AS<br>Jonas Lies vie 91<br>5021 Bergen, Norway
**Bioanalytical Pharmacokinetics Laboratory:**	ICON Development Solutions<br>8282 Halsey Rd<br>Whiteboro<br>NY 13492
**Clinical Research Organisation:**<br>(Monitoring, Data Management, Statistics & Programming, Medical Writing)	Ockham Oncology<br>Telephone: +44 131 200 6320
**Pharmacovigilance:**	Ockham Oncology<br>dsafety@ockham.com

3. Table of Contents

1. Protocol Approval Signatures 2
2. Study Personnel 3
3. Table of Contents 4
4. Tables and Figures 9
5. List of Abbreviations 10
6. Protocol Synopsis 13
6. Introduction 25
6.1. Pre-clinical Studies with BGB324 25
6.1.1. Summary of Pre-clinical Activity 25
6.1.2. Summary of Pre-clinical Toxicology 26
6.2. Clinical Studies with BGB324 27
6.2.1. Summary of Clinical Safety 27
6.2.1.1. ECG Findings 28
6.2.2. Summary of Clinical Pharmacokinetics 28
6.2.3. Selection of the Starting Dose for Clinical Study BGBC003 29
7. Study Objectives and Endpoints 30
7.1. Objectives 30
7.1.1. Primary Objectives 30
7.1.2. Secondary Objectives 31
7.1.3. Exploratory Objectives 31
7.2. Endpoints 31
7.2.1. Safety Endpoints 31
7.2.2. Efficacy Endpoints 31
7.2.2.1. AML 32
7.2.2.2. MDS 32
7.2.3. Pharmacokinetic Endpoints 32
7.2.4. Pharmacodynamic Endpoints 33
8. Study Design 33
8.1. Overview 33
8.2. Study Specifics 36
8.2.1. Phase I DLT Assessment Period 36
8.2.1.1. Dose Escalation Scheme 36

8.2.1.2. Dose Limiting Toxicity 38
8.2.1.3. Intra-Patient Dose Escalation in Part A 38
8.2.2. Part B 38
8.3. Overall Study Duration and Follow Up 38
8.3.1. Screening 38
8.3.2. Treatment 38
8.3.3. Final Study Visit 39
8.3.4. Follow Up 39
8.4. Study Stopping Rules 39
8.5. End of Study 40
9. Selection Of Subjects 40
9.1. Inclusion Criteria 40
9.2. Exclusion Criteria 40
10. Study Procedures and Assessments 42
10.1. Enrolment 53
10.1.1. Blinding Procedures 53
10.2. Screening 53
10.3. Treatment Period 54
10.3.1. Part A 54
10.3.1.1. Cycle 1 54
10.3.2. Cycle 2 56
10.3.3. Cycle 3 and Subsequent Cycles 57
10.3.4. Part B1 and Part B2 58
10.3.4.1. Cycle 1 58
10.3.5. Cycle 2 60
10.3.6. Cycle 3 and Subsequent Cycles 61
10.3.7. Part B3 61
10.3.7.1. Cycle 1 62
10.3.8. Cycle 2 64
10.3.9. Cycle 3 and Subsequent Cycles 65
10.3.10. Final Study Visit 66
11. Treatment Of Subjects 66
11.1. Treatment Schedule and Administration 66
11.1.1. BGB324 66

11.1.2. Ara-C 67
11.2. Dose Modification 67
11.2.1. BGB324 67
11.2.2. Ara-C 69
11.3. Concomitant Medication and Procedures 69
11.4. Continuation of Treatment 69
11.5. Discontinuation of Treatment and Withdrawal of Patients 69
12. Study Assessments 70
12.1. Patient Selection Assays 70
12.1.1. BGB324 Sensitivity Assay 70
12.1.2. Axl expression 70
12.2. Clinical Safety Assessments 70
12.2.1. Resting 12-lead ECG 71
12.2.2. Physical Examination 71
12.2.3. Vital signs 71
12.2.4. Subjective Symptomatology 71
12.3. Laboratory Safety Assessments 72
12.3.1. Haematology and Clinical Chemistry 72
12.3.2. Serum Pregnancy Testing 72
12.3.3. Urinalysis 73
12.4. Pharmacokinetic Assessments 73
12.4.1. Determination of BGB324 Concentration in Plasma 73
12.4.2. Determination of Ara-C Concentration in Plasma 73
12.5. Clinical Efficacy Assessments 73
12.6. Pharmacodynamic Assessments 74
12.6.1. Bone Marrow Aspirates 74
12.6.2. Blood Samples 74
12.6.3. Hair Follicle Samples 75
12.6.4. Normal Tissue Samples 75
12.7. Volume of Blood Sampling 75
13. Study Treatment Management 77
13.1. Study Medication 77
13.1.1. BGB324 77
13.1.1.1. BGB324 Storage 77

13.1.1.2. BGB324 Accountability 77
13.1.2. Ara-C 78
13.1.2.1. Ara-C Storage 78
13.1.2.2. Ara-C Accountability 78
14. Safety Definitions, Monitoring and Reporting 78
14.1. Definitions 78
14.1.1. Adverse Events 78
14.1.2. Adverse Reaction 79
14.1.3. Serious Adverse Events 79
14.2. Monitoring and Reporting Events 79
14.2.1. Adverse Events 79
14.2.2. Serious Adverse Events 79
14.2.3. All Events 80
14.3. Safety Classifications 80
14.3.1. Assessment of Severity 80
14.3.2. Assessment of Causality 81
14.3.3. Assessment of Expectedness 81
14.3.4. Immediate Reporting of Serious Adverse Events 81
14.3.5. Annual Safety Updates 82
14.3.6. Deaths 83
14.4. Procedures for Handling Special Situations 83
14.4.1. Pregnancy 83
14.4.2. Medical Emergency 83
14.4.3. Unblinding for a Medical Emergency 83
14.5. Investigator's Safety Responsibilities 83
14.6. Sponsor's Safety Responsibilities 84
15. Statistical Methods And Determination Of Sample Size 84
15.1. Description of Objectives 84
15.2. Description of Endpoints 84
15.3. Demography and Baseline Disease Characteristics 84
15.4. Safety 85
15.4.1. Analysis Population 85
15.4.2. Methods of Analysis 85
15.5. Efficacy 85

15.5.1. Analysis Population 85
15.5.2. Methods of Analysis 85
15.6. Pharmacokinetics 86
15.6.1. The Analysis Population 86
15.6.2. Methods of Analysis 86
15.7. Pharmacodynamics 86
15.7.1. The Analysis Population 86
15.7.2. Methods of Analysis 86
15.8. Safety Monitoring Committee 86
15.9. Sample Size Considerations 87
15.10. Clinical Study Report 87
16. Ethical Requirements 87
16.1. Good Clinical Practice Statement 87
16.2. Ethics Committee 88
16.3. Patient Information and Consent 88
16.4. Subject Data Protection 88
17. Administrative Procedures 89
17.1. Quality Assurance 89
17.2. Case Report Form and Source Document Verification 89
17.3. Study Monitoring 89
17.4. Notification of Serious Breaches 89
17.5. Retention of Study Data 90
17.6. Use of Information and Publication Policy 90
17.7. Contract Research Organisations 90
17.8. Changes to the Final Study Protocol 91
17.9. Investigator Responsibilities 91
18. References 92
19. Appendices 93

4. Tables and Figures

List of Tables

		Page
Table 1	BGB324 Kinase Selectivity Profile	25
Table 2	BGB324 –related Adverse Events Reported Following a Single Oral Administration of BGB324 (50 mg to 1500 mg) to Healthy Male Subjects	27
Table 3	Part A Schedule of Events	43
Table 4	Part B1 and B2 Schedule of Events	46
Table 5	Part B3 Schedule of Events	49
Table 6	Dose Modification of BGB324 for Toxicity	67
Table 7	Dose Modification of BGB324 for QTc Prolongation	68
Table 8	Haematology, Clinical Chemistry and Coagulation Parameters	72
Table 9	Urinalysis Parameters	73
Table 10	Volume of blood and BM aspirate to be drawn per Patient during Screening and Cycle 1	75
Table 11	Volume of Blood and BM aspirate to be drawn per Patient during Cycle 2	76
Table 12	Volume of Blood and BM aspirate to be drawn per Patient per Cycle $\geq$3 & Final Study Visit	76
Table 13	Plasma PK parameters	86
Table 14	Operating Characteristics of the 3+3 Study Design	87

List of Figures

		Page
Figure 1	Mean Plasma Concentrations of BGB324 Following a Single Oral Administration of BGB324 at 50, 100, 150, 200, 400, 600, 1000 and 1500 mg to Healthy Male Subjects	28
Figure 2	Simulated Plasma Concentrations of BGB324 Following Repeat Once Daily Oral Administration of 100 mg BGB324 to Healthy Male Subjects Under Fed Conditions (Loading Dose of 400 mg on Day 1 and Day 2)	30
Figure 3	BGBC003 Study Design	35
Figure 4	The 3+3 Dose Escalation Design	37

5. List of Abbreviations

ADR	Adverse reaction
AE	Adverse event
ALT	Alanine aminotransferase
ALP	Alkaline phosphatase
AML	Acute myeloid leukemia
ANC	Absolute neutrophil count
APTT	Activated Partial Thromboplastin Time
Ara-C	Cytosine arabinoside
AST	Aspartate aminotransferase
$AUC_{0\text{-}tau}$	Area under curve within a dosing interval
BM	Bone marrow
BP	Blood pressure
Ca	Calcium
Cl	Chloride
CK	Creatine Kinase
C_{max}	Maximum concentration achieved
CNS	Central nervous system
CR	Complete remission
CRi	CR with incomplete blood count recovery
CRF	Case report form
CRO	Contract Research Organisation
CSR	Clinical study report
CTCAE	Common Terminology Criteria for Adverse Events
CTD	Clinical Trial Directive
CV	Cardiovascular
DHA	Directions for Handling and Administration
DLT	Dose limiting toxicity
DSUR	Development Safety Update Report
ECOG	Eastern Cooperative Oncology Group
ECG	Electrocardiogram
EDC	Electronic data capture
EU	European Union
FCM	Flow cytometry
FDA	Food and Drug Administration
GCP	Good Clinical Practice
GMP	Good Manufacturing Practice
h	Hour

hERG	Human ether-à-go-go related gene
HIV	Human immunodeficiency virus
HPMC	Hydroxypropyl methylcellulose
HR	Heart rate
IB	Investigator’s Brochure
ICF	Informed consent form
ICH	International Conference on Harmonization
IHC	Immunohistochemistry
IMP	Investigational medicinal product
IND	Investigational new drug
IP	Inorganic phosphate
IPSS-R	Revised International Prognostic Scoring System
IRB	Institutional Review Board
IWG	International Working Group
K	Potassium
LC/MS/MS	Liquid chromatography/tandem mass spectrometry
MB	Myocardial B
MCH	Mean cell haemoglobin
MCHC	Mean corpuscular haemoglobin concentration
MDS	Myelodysplastic syndrome
MedDRA	Medical Dictionary for Regulatory Activities
MTD	Maximum tolerated dose
Na	Sodium
NCI	National Cancer Institute
NCI CTCAE	National Cancer Institute Common Terminology Criteria for Adverse Events
NYHA	New York Heart Association
ORR	Objective response rate
PD	Pharmacodynamic(s)
PBMC	Peripheral blood mononuclear cell(s)
PFS	Progression Free Survival
PK	Pharmacokinetic
PR	Partial remission
PII	Personally identifiable information
PR	Partial response
PT	Prothrombin time
QA	Quality Assurance
QTcF	QT interval utilising Fridericia’s correction
REC	Research Ethics Committee

RSI	Reference Safety Information
SAE	Serious adverse event
SAP	Statistical Analysis Plan
SAS	Safety analysis set
SMC	Safety Monitoring Committee
SmPC	summary of product characteristics
SPF	Sun Protection Factor
SUSAR	Suspected unexpected serious adverse reaction
$T_{½}$	Elimination half-life
T_{max}	Time to maximum concentration
TMF	Trial Master File
ULN	Upper limit of normal
WHO	World Health Organisation

6. Protocol Synopsis

PROTOCOL TITLE:	A Phase I multicenter open-label study of BGB324 as a single agent and in combination with cytarabine in patients with acute myeloid leukemia or high/intermediate (int-2) myleodysplastic syndrome which overexpresses Axl
PROTOCOL No:	BGBC003
SPONSOR:	BerGenBio AS<br>Norway
INVESTIGATIONAL PRODUCT:	BGB324
PHASE OF DEVELOPMENT:	Phase I
INDICATION AND RATIONALE:	Acute Myeloid Leukemia (AML) and high/intermediate (int-2) risk myelodysplastic syndrome MDS.<br>This is the first clinical study of BGB324 in patients.<br>BGB324 is a potent selective small molecule inhibitor of Axl, a surface membrane protein kinase receptor which is overexpressed in up to half of AML cases. Axl expression has been identified as a marker of a poor prognosis in AML. *In vitro* studies indicate that signalling through Axl stimulates a number of pro-survival pathways some of which are mediated by AKT phosphorylation and up-regulation of the epithelial receptor kinase pathway. *In vitro* studies indicate that activation of Axl enables malignant cells to develop resistance to conventional chemotherapies. Preliminary studies in AML cell lines indicate that treatment with BGB324 is at least additive in effect when administered with cytarabine (Ara-C).
OBJECTIVES:	**Part A**<br>PRIMARY<br>• To identify the maximum tolerated dose (MTD) of BGB324 in patients with relapsed or refractory AML or high risk MDS<br>SECONDARY<br>• To identify the dose limiting toxicity (DLT) profile of BGB324

	• To explore the safety and efficacy of BGB324<br>• To confirm the pharmacokinetic (PK) profile of BGB324<br>**Part B**<br>PRIMARY<br>• To identify the safety, tolerability and preliminary efficacy of BGB324, as a single agent and in combination with low dose Ara-C, in patients with newly diagnosed AML, who are unsuitable for intensive chemotherapy, or as a single agent in high/intermediate (int-2) risk MDS in a series of disease-specific cohorts<br>SECONDARY<br>• To explore the efficacy of BGB324 both alone and in combination with low dose Ara-C in patients with previously untreated AML, who are unsuitable for intensive chemotherapy, and as a single agent in patients with previously-treated high/intermediate (int-2) risk MDS<br>EXPLORATORY (Parts A and B)<br>• To demonstrate the pharmacodynamic activity of BGB324 by establishing its effects on relevant biological endpoints in bone marrow (BM) aspirates, peripheral blood mononuclear cells (PBMC) and hair follicles<br>• To investigate the pharmacodynamic activity of BGB324 in BM aspirates/PBMC (and a normal tissue control sample), by genomic analysis, to determine whether the spectrum of mutations present within the cancer cell population changes in response to treatment; to conduct a retrospective correlation with efficacy
ENDPOINTS:	SAFETY:<br>Clinically significant treatment emergent adverse events (AE), physical examination. Vital signs (i.e. blood pressure (BP) and heart rate (HR)), electrocardiogram (ECG), echocardiograms and laboratory findings including clinical chemistry,

	haematology and urinalysis.<br><br>EFFICACY:<br>• Objective response criteria:<br>o AML: Objective Response according to the revised recommendations of the International Working Group (IWG) in AML [Cheson et al. 2003]<br>o MDS: Objective Response according to the modified IWG response criteria in MDS [Cheson et al. 2006]<br>• Duration of Progression Free Survival (AML ONLY)<br><br>PHARMACOKINETIC:<br>As a minimum, area under the curve within a dosing interval ($AUC_{0\text{-}tau}$), maximum concentration achieved (C_{max}) and time to maximum concentration (T_{max}) for BGB324 (Part A and Part B3) and Ara-C (Part B3 only) will be determined from plasma. Additional parameters may also be reported, as deemed appropriate once data are reviewed.<br><br>PHARMACODYNAMIC:<br>The effects of BGB324 on pharmacodynamic endpoints of Axl inhibition will be determined in BM aspirates, blood samples and hair follicles:<br>• Effect of BGB324 on Axl and pAxl and downstream effectors of Axl signalling, to include but not limited to, Akt, pAkt, Erk, pErk, SLFN11, by appropriate methods (e.g. flow cytometry (FCM), immunohistochemistry (IHC), western blotting, proteomics, transcriptomics)<br>• Effect of BGB324 on gene expression by appropriate methods (e.g. FCM, IHC, western blotting, proteomics, transcriptomics, quantitative PCR, DNA-methylation analysis)<br>• To investigate the effect of BGB324 on

	the spectrum of mutations present within the cancer cell population by genomic analysis
STUDY DESIGN:	This Phase I open-label study will run at approximately seven clinical sites (in Germany, Norway and the US).<br>Up to 55 patients with AML or high/intermediate (int-2) risk MDS that overexpress Axl. In order to be eligible, patients with AML must have disease which is sensitive to BGB324 in *ex vivo* culture as defined by IC_{50} of $\leq$ 2.5 μM. Eligibility for MDS patients will be determined by BM blast surface membrane expression of Axl.<br>The study consists of a dose-escalation phase to determine the MTD of BGB324 in patients with relapsed or refractory AML or high risk MDS (Part A) followed by a cohort expansion phase in up to three disease-specific cohorts (Part B).<br>Dose-escalation will continue until DLT occurs, at which point an MTD will be selected and used in Part B.<br>BGB324 will be administered orally according to a daily schedule, with the first two doses of Cycle 1 serving as a 'loading' dose. Each 21-day (three week) period will constitute 1 cycle of treatment. The starting dose of BGB324 will be 400 mg on Cycle 1 Day 1 and Day 2, followed by a daily maintenance dose of 100 mg.<br><br>**Part A – Dose Escalation:**<br>Open label, dose-escalation study of repeat doses of BGB324 as a single agent. Escalation will be performed according to a "3+3" design to determine the MTD. A minimum of 10 patients will be treated at the MTD.<br>The DLT assessment period will comprise the first 21-day treatment cycle. Patients who do not complete the DLT assessment period for reasons other than toxicity will be replaced for the purpose of toxicity evaluation. A patient must receive $\geq$9 of the 10 doses in Cycle 1 in order to be considered evaluable for toxicity.<br>The decision to dose-escalate (or not) will be made by a Safety Monitoring Committee (SMC)

	comprising a representative from each actively recruiting investigational site and a representative of the BerGenBio AS study team. The decision to dose escalate will be based upon the tolerability of BGB324 observed at the previous dose level. Dose escalation will range between 25 and 50% of the previous dose according to the nature and severity of the toxicity observed.<br>If the starting dose of 400 mg loading/100 mg daily maintenance exceeds the MTD, a lower dose may be explored if recommended by the SMC.<br>To optimize the chance of each patient receiving therapeutically optimal doses of BGB324 up to a maximum of two intra-patient dose escalations will be permitted as long as no CTCAE ≥Grade 2 non-hematological toxicities unrelated to disease progression, inter-current illness or concomitant medication occurred at the lower dose. Once the MTD has been identified, no intra-subject dose-escalation may occur above that dose.<br><br>**Part B – Cohort Expansion**<br>Up to three distinct patient cohorts of 14 patients per cohort will be explored depending upon the efficacy and toxicity of BGB324 observed in Part A. The decision to perform individual cohorts will also be dependent upon emerging biological data.<br>Part B1: Single agent BGB324 in patients with intermediate (int-2) or high risk MDS, according to International Prognostic Scoring System (IPSS) Risk Stratification (Greenberg et al. 1997), who have received no more than a single line of demethylating therapy.<br>Part B2: Single agent BGB324 in newly diagnosed patients with AML who are not suitable for intensive chemotherapy.<br>Part B3: In combination with low dose Ara-C in newly diagnosed patients with AML who are not suitable for intensive chemotherapy.<br>BGB324 will be administered at the MTD in Parts B1 and B2.<br>In Part B3 BGB324 will be administered at a

	dose identified by the SMC in combination with Ara-C administered according to standard practice (i.e. 10 mg/m^2 twice daily for 14 days followed by a rest period of $\leq$1 month according to persisting myelosuppression). If the dose of BGB324 is less than the single agent MTD further dose escalation of BGB324 may be considered.
DOSE LIMITING TOXICITIES (Part A)	DLT will be evaluated during the first three weeks (one cycle) of treatment and include:<br>• CTCAE Grade 3 or 4 nausea, vomiting, or diarrhea despite maximum prophylactic and supportive care<br>• Any other CTCAE Grade 3 or 4 non-hematological toxicity that is considered to be clinically significant and causally related to BGB324<br>• Treatment discontinuation or dose reduction for more than 72 hours as a result of BGB324-related toxicity<br>• Pancytopenia with a hypocellular bone marrow (≤5% cellularity) and no evidence of leukemia, lasting longer than 42 days
STUDY POPULATION:<br>PRINICPAL INCLUSION CRITERIA	1. Provision of signed written informed consent<br>2. Histological or cytological confirmation of one of the following:<br>a) AML which is sensitive to BGB324 *ex vivo* as defined by an IC_{50} of $\leq$2.5 μM<br>b) Part A: High risk group MDS, according to IPSS Risk Stratification, expressing Axl by FCM<br>c) Part B: High/intermediate (int-2) risk group MDS, according to IPSS Risk Stratification, expressing Axl by FCM<br>3. Patients with AML:<br>a) Relapsed or refractory disease (Part A)<br>b) Newly diagnosed who are unsuitable for intensive chemotherapy (Part B)<br>4. European Cooperative Oncology Group (ECOG) performance status 0, 1 or 2 [Appendix 1]<br>5. Male or female, age 18 years or older<br>6. Female patients of childbearing potential must have a negative serum pregnancy

	test within 3 days prior to taking their first dose of BGB324. Male patients and female patients of reproductive potential must agree to practice highly effective methods of contraception (such as hormonal implants, combined oral contraceptives, injectable contraceptives, intrauterine device with hormone spirals, total sexual abstinence, vasectomy) throughout the study and for ≥3 months after the last dose of BGB324. Female patients are considered NOT of childbearing potential if they have a history of surgical sterility or evidence of post-menopausal status defined as any of the following:<br>a) Natural menopause with last menses >1 year ago<br>b) Radiation induced oophorectomy with last menses >1 year ago<br>c) Chemotherapy induced menopause with last menses >1 year ago
PRINCIPAL EXCLUSION CRITERIA:	1. Pregnant or lactating<br>2. Abnormal left ventricular ejection fraction on echocardiography (less than the lower limit of normal for a patient of that age at the treating institution)<br>3. History of an ischaemic cardiac event including myocardial infarction within 3 months of study entry<br>4. Congestive cardiac failure of > Grade 2 severity according to the NYHA [Appendix 2] as defined as symptomatic at less than ordinary levels of activity<br>5. Unstable cardiac disease including unstable angina or hypertension as defined by the need for change in medication within the last three months<br>6. History or presence of bradycardia (less than or equal to 60 BPM) or history of symptomatic bradycardia, left bundle branch block, cardiac pacemaker or significant atrial tachyarrythmias<br>7. Current treatment with any agent that may prolong QT interval and may cause Torsade de Points [Appendix 3] which cannot be discontinued at least two

	weeks prior to treatment<br>8. Known family or personal history of long QTc syndrome or ventricular arrhythmias including ventricular bigeminy<br>9. Previous history of drug-induced QTc prolongation<br>10. Screening 12-lead ECG with a measurable QTc interval according to Fridericias correction > 450 ms<br>11. Abnormal serum potassium, calcium or magnesium levels according to the local laboratory<br>12. Ongoing infection requiring systemic treatment<br>13. Inadequate liver function as demonstrated by serum bilirubin ≥1.5 times the upper limits of normal range (ULN) or alanine aminotransferase (ALT) or aspartate aminotransferase (AST) ≥2.5 times the ULN (or ≥5 times the ULN in the presence of liver involvement by leukemia)<br>14. Inability to tolerate oral medication<br>15. Existing gastrointestinal disease affecting drug absorption such as celiac disease or Crohns disease<br>16. Previous bowel resection<br>17. Evidence of ongoing gastrointestinal graft versus host disease<br>18. Haematopoetic stem cell transplantation within 6 months<br>19. Impaired renal function as demonstrated by creatinine ≥1.5 times the ULN or creatinine clearance of ≤50 mL/min determined by Cockcroft-Gault formula<br>20. Radiotherapy or chemotherapy within the 14 days prior to the first dose of BGB324 being administered (other than hydroxyurea)<br>21. Receiving an investigational anti-cancer treatment concurrently or within 14 days or five half-lives of either the parent drug or any active metabolite prior to the start of BGB324<br>22. Unresolved CTCAE Grade 2 or greater toxicity (other than stable toxicity) from previous anti-cancer therapy excluding

	alopecia<br>23. Any evidence of severe or uncontrolled systemic conditions (e.g., severe hepatic impairment) or current unstable or uncompensated respiratory or cardiac conditions which makes it undesirable for the patient to participate in the study or which could jeopardize compliance with the protocol<br>24. Active, uncontrolled central nervous system (CNS) disease including CNS leukemia<br>25. Active infection with human immunodeficiency virus (HIV), hepatitis B or C viruses – screening for viral infections is **not** required for entry to this study<br>26. Major surgery within 28 days prior to the start of BGB324 – excluding skin biopsies and procedures for insertion of central venous access devices<br>27. Known hypersensitivity to cytarabine (Ara-C) or its excipients
TREATMENT AND INTERVENTIONS	Patient eligibility for the study will be determined within 14 days prior to the first dose of BGB324. Screening assessments will be conducted according to the Schedule of Events (Tables 3-5) in Section 10.<br>Eligible patient will visit the study site to receive study treatment and protocol-specified procedures according to the relevant Schedule of Events (Tables 3-5) in Section 10.<br>The treatment period will consist of continuous 21-day treatment cycles. Patients will be intensively monitored throughout Day 1 to Day 4 inclusive of Cycle 1, and in addition will attend the clinic on Day 8, and Day 15. Patients will attend the clinic once a week during Cycle 2 (Day 1, 8 and 15) and then once per cycle thereafter.<br>The study period will consist of screening, treatment, Final Study Visit, and follow-up. The Final Study Visit will occur 28 days after the patient has discontinued study treatment.
BGB324:FORMULATION/DOSE/ROUTE OF ADMINISTRATION:	BGB324 is presented for oral dosing in size zero capsules each containing 100 mg of drug

	substance.<br>BGB324 will be administered according to a daily dosing schedule during each week of continuous 21-day treatment cycles. During Cycle 1 ONLY the first two doses of Cycle 1 comprise a 'loading' dose.<br>**Part A**: Dose is ascending per dose cohort. The starting dose will be 400 mg on Cycle 1 Day 1 and Day 2, followed by a daily maintenance dose of 100 mg.<br>**Part B:** BGB324 will be administered at the MTD in Parts B1 and B2. In Part B3 BGB324 will be administered at a dose identified by the SMC in combination with Ara-C administered according to standard practice (i.e. 10 mg/m^2 twice daily for 14 days followed by a rest period of $\leq$1 month according to persisting myelosuppression).
DURATION AND FREQUENCY	Patients may continue to receive BGB324 for as long as, in the opinion of the investigator, they continue to derive clinical benefit or until disease progression, development of severe toxicity, or withdrawal of consent.<br>Patients who discontinue treatment with Ara-C in Part B3 may continue to receive BGB324 alone.<br>Patients with AML who are receiving BGB324 as monotherapy and do not achieve an objective response following two cycles of treatment with BGB324 may start additional treatment with orally administered anti-metabolites (e.g. 6-mercaptopurine, hydroxyurea), melphalan or low dose Ara-C from Cycle 3.
EVALUATION:<br>Safety:<br>Physical examination<br>Vital signs<br>ECG<br>Echocardiogram<br>Routine blood panel (haematology, clinical chemistry and coagulation panel)<br>Adverse events (CTCAE)<br><br>Efficacy:<br>Peripheral blood count; BM aspirate	As outlined in the relevant Schedule of Events (Tables 3-5)

Pharmacokinetic:<br>BGB324 (Part A and Part B3) and Ara-C (Part B3 only) PK parameters in plasma<br><br>Pharmacodynamic:<br>The effects of BGB324 on pharmacodynamic endpoints, gene expression and genomics in BM aspirates, PBMC, hair follicles and skin biopsy/hair samples	
FOLLOW UP	AEs will be monitored for 28 days after the last dose of BGB324. If BGB324-related toxicities continue beyond the follow up period, patients will be followed until all BGB324-related toxicities have resolved, stabilised or returned to baseline. Follow up monitoring for AEs may be conducted over the telephone.<br>The study will be completed when all patients have completed their final study visit. Further information on disease progression and overall survival may be collected and included as an addendum to the clinical study report.<br>Patients who stop treatment with BGB324 in Part B3 but remain on treatment with Ara-C will be considered as having discontinued the study.
STATISTICAL METHODS	Only descriptive analysis will be performed on safety data - no formal statistical testing will be conducted. Although all efficacy analysis is to be regarded as exploratory, there will be formal hypothesis tests for the objective response rates within the MTD cohort.<br>**Safety**<br>All safety and tolerability assessments will be based on the safety analysis set (all patients who have received at least one dose of study medication).<br>**Efficacy**<br>Efficacy assessments will be based on the safety analysis set<br>**Pharmacokinetics**<br>Where possible, individual, mean and median BGB324 plasma concentration-time data will be presented in tabular and/or graphical form. As a

	minimum, area curve within a dosing interval ($AUC_{0\text{-}tau}$), maximum concentration achieved (C_{max}), time to maximum concentration (T_{max}) and elimination half-life ($T_{½}$), for BGB324 and Ara-C (Part B3 only) will be determined.<br>**Pharmacodynamics**<br>Pharmacodynamic data will be listed and summarized by dose cohort and time point as appropriate.

6. Introduction

This study is a dose-escalation and cohort expansion study of BGB324, an axl kinase inhibitor, in patients with acute myeloid leukemia (AML) and myelodysplastic syndrome (MDS), who overexpress axl, either as a single agent or in combination with cytosine arabinoside (Ara-C). Despite current therapies the majority of adult patients with AML and high/intermediate (int-2) risk MDS continue to die from their disease and thus their need for new therapies remains high and access to novel, targeted therapies may provide clinical benefit.

6.1. Pre-clinical Studies with BGB324

6.1.1. Summary of Pre-clinical Activity

BGB324 demonstrates potent inhibition of Axl in biochemical and cell-based kinase inhibition assays. The selectivity of BGB324 for Axl is illustrated in Table 1.

Table 1: BGB324 Kinase Selectivity Profile

Kinase	Kinome Scan binding assay (Kd)		KinaseProfiler kinase activity assay (IC_{50})		BaF3 cell-based kinase activity assay (IC_{50})	
	nM	fold	nM	fold	nM	fold
Axl	0.4	1	4.6	1	63	1
Tie2	270	680	30	6.4	355	5.5
Ret	73	180	38	8.1	>316	>5
Flt1	400	>1000	40	8.7	>1000	>15
Flt4	460	>1000	41	8.8	>1000	>15
Yes	810	>1000	43	9.2	n/a	n/a

n/a = not applicable

BGB324 inhibits the growth and survival of tumor cell lines derived from a range of solid and leukemic tumors. *In vitro* activity of BGB324 has been examined in a range of different AML cell lines where exposure-induced differentiation and arrest in the G1 phase of the cell cycle was accompanied by a reduction in levels of phosphorylated axl and phosphorylated ERK.

The *in vivo* efficacy of BGB324 has been evaluated in two different murine models of AML. Studies in the MDV4-11 xenograft model showed a dose-dependent reduction in tumor burden following oral treatment with BGB324. A dose-dependent reduction in both Axl and ERK phosphorylation levels were noted. BGB324 treatment also significantly inhibited tumor growth in the OCI-AML5 xenograft model. Furthermore BGB324 has been shown to significantly prolong survival in the syngeneic bone marrow transplantation Hoxa9/Meis1 mouse model [*Ben-Batalla et al., 2013*].

As described by Ben-Batalla *et al.,* bone marrow cells from 7 out of 11 patients with primary AML were sensitive to BGB324 (IC_{50} = 1.9 ± 0.5μM), and this inhibition was independent of FLT3 status since FLT3 WT AML cells were similarly sensitive to BGB324 (n=3/8; p=0.4212). Furthermore, the EC_{50} of response to BGB324 correlated with Axl protein expression levels (n=10; R^2=0.84; p<0.05), with Axl negative cells being almost completely resistant to BGB324. BGB324 treatment also significantly sensitizes bone marrow cells from primary patient AML cells to Ara-C [*Ben-Batalla et al., 2013*].

Population studies have identified both Axl expression and circulating levels of Gas 6 as independent predictors of prognosis [*Whitman et al., 2013*].

A modest substrate and time-dependent inhibition of CYP3A4/5 activity by BGB324 was observed in an *in vitro* study with human liver microsomes. Preliminary results from an Ara-C *in vitro* metabolism study indicate that Ara-C does not undergo extensive metabolism by CYP3A4. Consequently it is considered unlikely that there will be an interaction between BGB324 and Ara-C.

A comprehensive summary of the pre-clinical activity of BGB324 in AML is presented in the Investigator's Brochure (IB) version 5.0.

6.1.2. Summary of Pre-clinical Toxicology

To support clinical studies with BGB324 a series of animal toxicology and safety studies, including 28-day repeat dosing studies in rodents and monkeys and a single dose telemetered cardiovascular (CV) safety study in monkeys, have been conducted. Comparisons of the data reported in the rodent and monkey studies indicate that primates are more sensitive to BGB324 on a per body weight basis.

The results of the repeat dose 28-day study in monkeys identified the liver, reticuloendothelial system and haematopoietic system as target organs. These events were largely thought to be due to macrophage accumulation, consistent with phospholipidosis, and were only partially reversible across the planned 16 day recovery period, although this was anticipated to be due to the slow elimination of the compound. Similar findings were identified in mice treated across a range of dose levels with partial recovery noted 16 days later.

In the CV safety study, single oral administration of BGB324 (7.5 mg/kg – 60 mg/kg) was associated with recoverable, non-adverse decreases in heart rate and corresponding increases in the RR interval durations. Dose-dependent increase in QTc interval was noted at all dose levels; the magnitude of the QTc effect at the highest dose level was 17%, whilst at 7.5 mg/kg and 15 mg/kg, the change generally fell below 10% compared to vehicle control

and pre-dose values. These observations were consistent with the previously identified IC_{50} for human ether-à-go-go related gene hERG channel inhibition of 0.53 μM BGB324.

BGB324 was negative in the Ames bacterial mutation assay, suggesting that BGB324 is not mutagenic. BGB324 was also negative in an *in vitro* mammalian cell cytogenetic study in human lymphocytes suggesting that BGB324 is not clastogenic.

6.2. Clinical Studies with BGB324

There has been one previous clinical study conducted with BGB324 (Protocol BGBC001). This study explored the effect of a single dose of BGB324 in healthy male volunteers. Eight dose levels (50 mg, 100 mg, 150 mg, 200 mg, 400 mg, 600 mg, 1000 mg and 1500 mg) were administered under fasted conditions to cohorts of 4 subjects (32 subjects in total). Seven of these subjects went on to receive the same dose of BGB324 administered under fed conditions.

6.2.1. Summary of Clinical Safety

In general exposure to BGB324 was well tolerated with all toxicities being spontaneously reversible and predominantly gastrointestinal in nature. No serious adverse events (SAE) were reported. Reported adverse events (AE) are summarised in Table 2.

Table 2: BGB324-Related Adverse Events Reported Following a Single Oral Administration of BGB324 (50 mg to 1500 mg) to Healthy Male Subjects

Dose (mg)	Number of Subjects	Toxicity	Maximum Grade (CTCAE version 4.0)
50	4 fasted; 1 fed	Non-Cardiac Chest Pain	One
100	4 fasted; 2 fed	Orthostatic hypotension	One
150	4 fasted; 1 fed	Abdominal Distension x 2	One
400	4 fasted; 2 fed	Diarrhoea	One
600	4 fasted	Diarrhoea<br>Flatulence<br>Nausea x 2	One<br>One<br>One
1000	4 fasted	Nausea	One
1500	4 fasted	Diarrhoea<br>Nausea<br>Vomiting<br>Headache<br>Dizziness	One<br>Two<br>Two<br>One<br>One

6.2.1.1. ECG Findings

In light of the findings reported in the safety pharmacology studies described in Section 6.1.2, all subjects in study BGBC001 underwent serial ECG recordings following the administration of BGB324. The overall pattern indicated that BGB324 administration was accompanied by a variable increase in QTcF, the magnitude of which was related to systemic exposure. The maximum QTcF recorded was Grade I in severity.

6.2.2. Summary of Clinical Pharmacokinetics

Concentrations of BGB324 were quantified in plasma samples from subjects enrolled in Study BGBC001 using a fully validated liquid chromatography/tandem mass spectrometry (LC/MS/MS) method with a LLOQ of 2.0 ng/mL. PK parameters were derived by non-compartmental analysis using WinNonlin Phoenix Version 6.3 (Pharsight Corporation Inc., Mountain View, CA, USA, 2012).

Following a single oral dose of BGB324 at doses between 50 mg and 1500 mg, administered under fasted conditions, mean maximal plasma concentrations of 7.63 ng/mL to 388 ng/mL were reached at 6 hours (h) to 23 h post-dose as presented in Figure 1.

Figure 1: Mean Plasma Concentrations of BGB324 Following a Single Oral Administration of BGB324 at 50, 100, 150, 200, 400, 600, 1000 and 1500 mg to Healthy Male Subjects

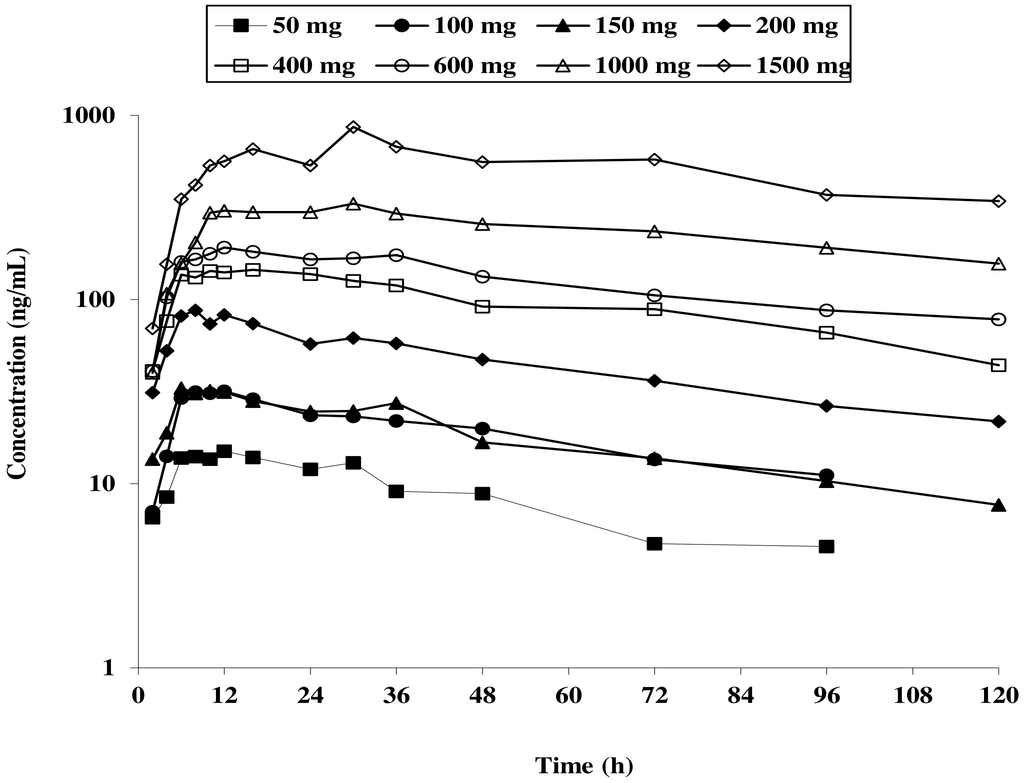

The mean apparent terminal half-life ($t_{1/2}$) of BGB324 was 59.8 h to 92.1 h. Between-subject variability in systemic exposure (AUC_{0-t} and C_{max}) to BGB324 at 50 mg to 1500 mg was generally high (CV of 25.8% to 97.0% for AUC_{0-t} and C_{max} respectively), most probably reflecting variable absorption. Across this dose range C_{max} of BGB324 increased approximately dose proportionately, although the AUC_{0-t} of BGB324 increased slightly greater than dose proportionately.

Following administration of BGB324 with food (whole milk and buttered toast) to 7 subjects who had previously received BGB324, there was an overall reduction in the between-subject variability with an increase in systemic exposure to BGB324 in 3 subjects, no appreciable change in 4 subjects and an apparent reduction in 1 subject. It was estimated that administration with food would achieve an increase in exposure in some subjects of up to two fold.

6.2.3. Selection of the Starting Dose for Clinical Study BGBC003

In healthy subjects who received a single oral dose of BGB324 (Study BGBC001) the elimination of BGB324 was slow with a $t_{1/2}$ of longer than 24 hours. The administration schedule and starting of BGB324 proposed for Study BGBC003 is based on fitting a one compartment model, incorporating a lag time and no weighting, to the available clinical PK data. It is anticipated that administration of a loading dose of BGB324 (on Day 1 and Day 2) followed by daily dosing will deliver the optimum exposure of BGB324 to patients enrolled in Study BGBC003. The application of a loading dose will enable a rapid achievement of therapeutic levels, whilst daily dosing will prevent wide changes in systemic concentration during therapy. The simulated mean plasma concentration-time profile of BGB324, based on the PK model, following dosing of BGB324 at 400 mg on Day 1 and Day 2 and 100 mg daily thereafter is presented in Figure 2. BGB324 will be administered with food as preliminary evidence indicates that this approach may maximise bioavailability.

Figure 2: Simulated Plasma Concentrations of BGB324 Following Repeat Once Daily Oral Administration of 100 mg BGB324 to Healthy Male Subjects Under Fed Conditions (Loading Dose of 400 mg on Day 1 and Day 2)

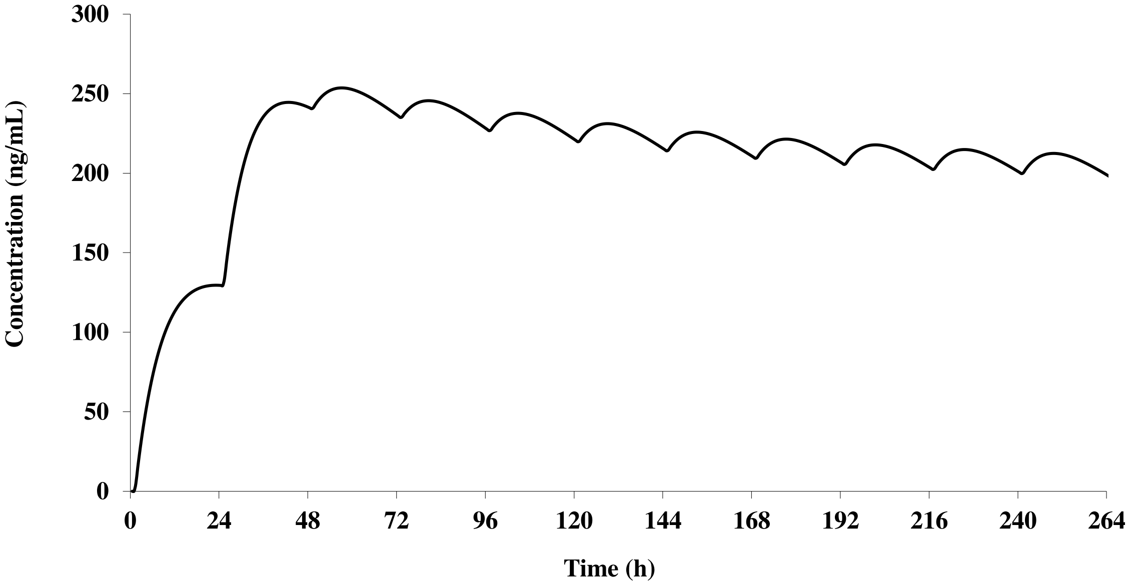

On the basis of the PK profile already identified in healthy subjects (Study BGBC001), it is expected that a loading dose of 400 mg (Day 1 and Day 2) followed by 100 mg daily will achieve an initial circulating concentration of approximately 250 ng/mL on Day 2/3 declining to a steady state value of 200 ng/mL thereafter. According to the extensive PK-ECG modelling performed on the data collected in healthy subjects, a plasma concentration of 250 ng/mL would be expected to result in a mean increase in QTcF of 38 ms from baseline falling to 32 ms from baseline at the anticipated steady state concentration of 200 ng/mL. This anticipated level of increase is considered acceptable in a closely monitored population excluding patients at high risk of ventricular arrhythmias. Further dose escalation will only be considered following review of the ECG changes observed in patients treated at this dose level. Please refer to Table 7 in Section 11.2 for guidance on dose modification on the basis of QTc changes..

7. Study Objectives and Endpoints

7.1. Objectives

7.1.1. Primary Objectives

Part A

To identify the maximum tolerated dose (MTD) of BGB324 in patients with relapsed or refractory AML or high risk MDS.

Part B

To identify the safety, tolerability and preliminary efficacy of BGB324, as a single agent and in combination with low dose Ara-C, in patients with newly diagnosed AML who are unsuitable for intensive chemotherapy, or as a single agent in high/intermediate (int-2) risk MDS in a series of disease-specific cohorts.

7.1.2. Secondary Objectives

Part A

- To identify the Dose Limiting Toxicity (DLT) profile of BGB324
- To explore the safety and tolerability of BGB324
- To confirm the pharmacokinetic (PK) profile of BGB324

Part B

- To explore the efficacy of BGB324 both alone and in combination with low dose Ara-C in patients with previously untreated AML, who are unsuitable for intensive chemotherapy and as a single agent in patients with previously-treated high/intermediate (int-2) risk MDS

7.1.3. Exploratory Objectives

Parts A and B

To demonstrate the pharmacodynamic (PD) activity of BGB324 by establishing its effects on relevant biological endpoints in bone marrow (BM) aspirates, peripheral blood mononuclear cells (PBMC) and hair follicles

7.2. Endpoints

7.2.1. Safety Endpoints

Safety and tolerability will be assessed by conducting the following safety assessments at pre-defined time-points during the study:

- Clinically significant treatment emergent AEs
- Physical examination
- Vital signs including blood pressure (BP), heart rate (HR)
- Electrocardiogram (ECG)
- Echocardiogram
- Laboratory findings including clinical chemistry, hematology and urinalysis

7.2.2. Efficacy Endpoints

Efficacy endpoints will be measured at a minimum every three weeks prior to administering study medication on the second and subsequent cycles using objective response criteria.

7.2.2.1. AML

- Objective Response according to the revised recommendations of the International Working Group (IWG) in AML [*Cheson et al., 2003*]:
  - Morphologic Complete remission (CR) <u>all</u> of the following must be achieved:
    - Morphological leukemia-free state:
      - ≤ 5% blasts in the bone marrow (BM) aspirate
    - Absolute neutrophil count (ANC) ≥1.0 x 10^9/L
    - Platelet count ≥100 x 10^9/L
  - Morphologic CR with incomplete blood count recovery (CRi):
    - Morphological leukemia-free state:
      - ≤ 5% blasts in the BM aspirate
    - ANC ≥1.0 x 10^9/L
  - Partial remission (PR)
    - Peripheral blood count recovery as for CR
    - Decrease of ≥50% in the percentage of blasts (to 5 - 25% blasts) in the BM aspirate
- Duration of Progression Free Survival (PFS)

7.2.2.2. MDS

- Objective Response according to the modified IWG response criteria in MDS [*Cheson et al., 2006*]:
  - Morphologic CR <u>all</u> of the following must be achieved:
    - ≤5% myeloblasts in the BM aspirate
    - 0% blasts
    - ANC ≥1.0 x 10^9/L
    - Platelet count ≥100 x 10^9/L
    - Hemoglobin ≥11g/dL
  - PR
    - As for CR, but bone marrow blasts decreased by ≥50% over pre-treatment value but still >5%

7.2.3. Pharmacokinetic Endpoints

As a minimum, area under the curve within a dosing interval ($AUC_{0\text{-}tau}$), maximum concentration achieved (C_{max}), and time to maximum concentration (T_{max}) for BGB324 (Part A and Part B3) and Ara-C (Part B3) will be determined from plasma. Additional parameters may also be reported, as deemed appropriate once data are reviewed.

7.2.4. Pharmacodynamic Endpoints

The effects of BGB324 on pharmacodynamic endpoints of Axl inhibition will be determined in BM aspirates, PBMC samples and hair follicles. Methods such as IHC, western blotting, ELISA, FCM, quantitative PCR, DNA-methylation analysis, transcriptomics, proteomics and genomics will be utilised.

- Effect of BGB324 on Axl and pAxl and downstream effectors of Axl signalling, to include but not limited to, Akt, pAkt, Erk, pErk, SLFN11, by appropriate methods (e.g. FCM, IHC, western blotting, proteomics, transcriptomics)
- Effect of BGB324 on gene expression by appropriate methods (e.g. transcriptomics, DNA-methylation analysis, proteomics, quantitative PCR, FCM, IHC, western blotting)
- Genomic analysis of normal tissue, and of cancer cells prior to and during treatment with BGB324, to investigate whether the spectrum of mutations present within the cancer cell population changes with BGB324 treatment

8. Study Design

8.1. Overview

This is a phase Ib, open-label, dose-escalation and cohort expansion study to obtain a preliminary assessment of the safety, tolerability and efficacy of BGB324, a small molecule inhibitor of Axl kinase in patients with acute AML or high/intermediate (int-2) risk MDS that overexpress Axl. BGB324 will be administered as monotherapy in relapsed or refractory AML and MDS patients and as a monotherapy or in combination with Ara-C in previously untreated AML patients.

This study will be performed in approximately 55 patients with acute AML or high/intermediate (int-2) risk MDS. In order to be eligible, patients with AML must have disease which is sensitive to BGB324 in ex vivo culture as defined by IC_{50} of $\leq$2.5 μM. Eligibility for MDS patients will be determined by surface membrane expression of Axl on BM blasts by FCM.

BGB324 will be administered orally according to a daily schedule during continuous 21-day treatment cycles. During Cycle 1, Week 1 ONLY the first two doses of BGB324 will serve as a 'loading' dose. The starting dose of BGB324 will be 400 mg on Cycle 1 Day 1 and Day 2 (loading dose) followed by a daily maintenance dose of 100 mg.

In Part A the study will follow a 3+3 design to explore the safety and tolerability of repeat dosing of BGB324, and to identify the MTD. A total of 10 patients will be treated at the MTD. Depending upon the results of this phase of the study, the safety and efficacy of BGB324

may be established in up to 3 disease-specific cohorts in Part B comprising 14 patients each.

Part B1

Single agent BGB324 will be administered to patients with intermediate (int-2) or high risk MDS, according to the International Prognostic Scoring System (IPSS) Risk Stratification [*Greenberg et al., 1997*], who have received no more than a single line of demethylating therapy.

Part B2

Single agent BGB324 will be administered to patients with newly diagnosed AML who are not suitable for intensive chemotherapy.

Part B3

BGB324 will be administered in combination with Ara-C (at a dose of 10mg/m^2 administered subcutaneously twice daily) to patients with newly diagnosed AML who are not suitable for intensive chemotherapy.

The study will be conducted at approximately 7 clinical sites in Germany, Norway and the United States.

Please refer to Figure 3 for an outline of the overall study design.

Figure 3: BGBC003 Study Design

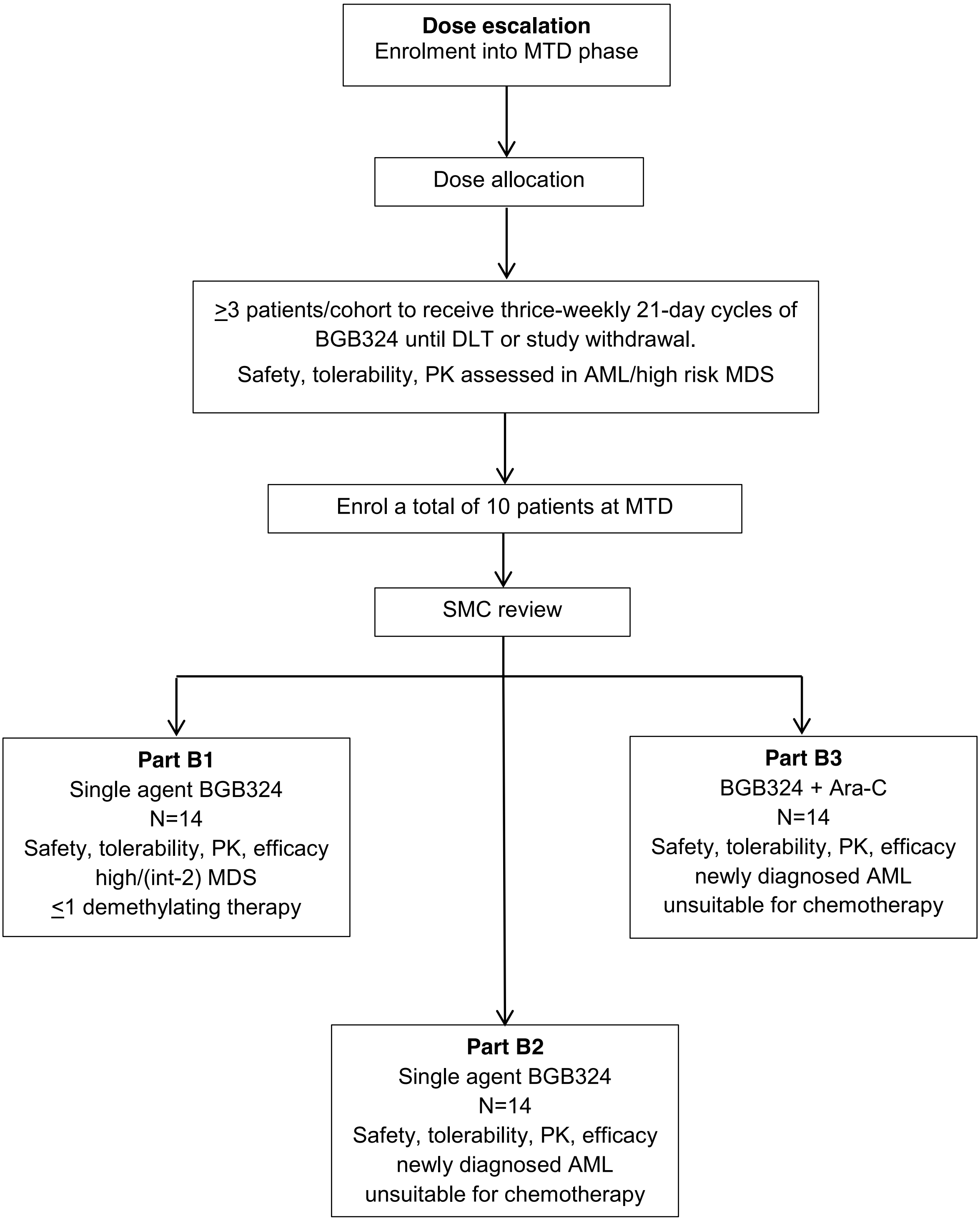

8.2. Study Specifics

8.2.1. Phase I DLT Assessment Period

During the dose-escalation phase (Part A), safety, tolerability, PK, and preliminary clinical activity of BGB324 will be assessed in patients with relapsed or refractory AML or high risk MDS and the MTD will be established.

The starting dose (Cohort 1) will be 400 mg on Cycle 1 Day 1 and Day 2, followed by a daily maintenance dose of 100 mg during continuous 21-day treatment cycles. At least three evaluable patients will be entered per cohort.

8.2.1.1. Dose Escalation Scheme

Initially three patients will be recruited into Cohort 1 and will complete 1 cycle of treatment before a dose-escalation evaluation is made. A patient must receive ≥9 of the 10 doses in Cycle 1 in order to be considered evaluable for toxicity.

The decision to dose-escalate (or not) will be made by a Safety Monitoring Committee (SMC) comprising a representative from each actively recruiting investigational site and a representative of the BerGenBio AS study team. Minutes of the SMC meeting will be recorded and circulated for final approval before being placed in the Trial Master File (TMF). The decision to dose escalate will be based upon the tolerability of BGB324 observed at the previous dose level. Dose escalation will range between 25 and 50% of the previous dose according to the nature and severity of the toxicity observed.

If dose escalation occurs, three patients will be recruited to Cohort 2 and will complete 1 cycle of treatment before a dose-escalation evaluation is made, as outlined above.

If one patient in a cohort experiences a DLT during Cycle 1, the cohort will be expanded to 6 patients. If two of 3 or 2 of 6 patients in a cohort experience DLT no further dose-escalation will take place and the dose below will be nominated as the MTD. If the starting dose of 400 mg loading/100 mg daily maintenance exceeds the MTD, a lower dose may be explored if recommended by the SMC.

If a patient withdraws or is withdrawn for reasons other than DLT prior to completing Cycle 1, the patient will be replaced.

Please refer to Figure 4 for the operating characteristics of the Dose Escalation process.

Figure 4: The 3+3 Dose Escalation Design

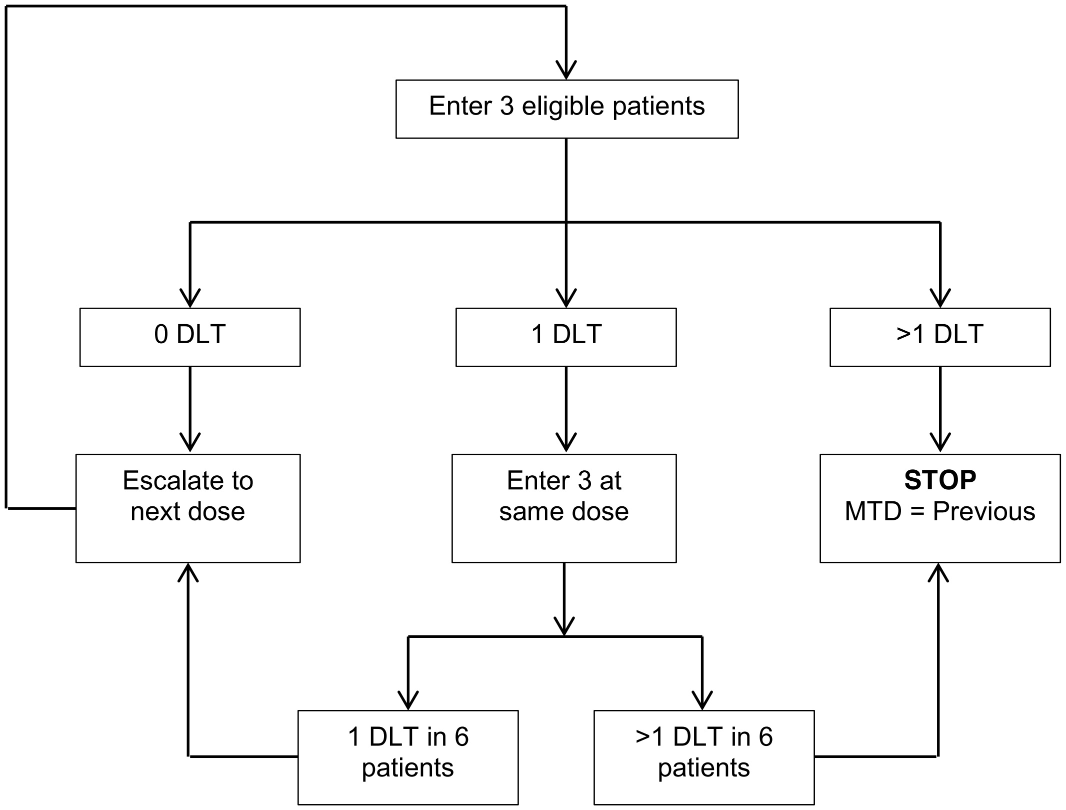

8.2.1.2. Dose Limiting Toxicity

DLT will be assessed during the first 3 weeks of treatment (Cycle 1), according to the National Cancer Institute (NCI) Common Terminology Criteria for Adverse Events [NCI CTCAE] version 4, and is defined as any one of the following

- CTCAE Grade 3 or 4 nausea, vomiting, or diarrhea despite maximum prophylactic and supportive care
- Any other CTCAE Grade 3 or 4 non-hematological toxicity that is considered to be clinically significant and causally related to BGB324. Isolated changes in laboratory results that have no clinical sequelae and are <1 week in duration are not considered dose limiting
- Treatment discontinuation or dose reduction for >72 hours as a result of BGB324-related toxicity
- Pancytopenia with a hypocellular bone marrow (≤5% cellularity) and no evidence of leukemia lasting longer than 42 days

8.2.1.3. Intra-Patient Dose Escalation in Part A

After a patient has completed their DLT assessment period, and in order to optimize their chance of receiving therapeutically optimal doses of BGB324, up to a maximum of 2 intra-patient dose escalations will be permitted as long as no CTCAE ≥Grade 2 non-hematological toxicities unrelated to disease progression, inter-current illness or concomitant medication occurred at the lower dose. Once the MTD has been identified, no intra-subject dose-escalation may occur above that dose.

8.2.2. Part B

Following SMC review of all treatment emergent safety and efficacy data from Part A, fourteen patients will be recruited into each of up to 3 cohorts (Parts B1, B2 and B3).

8.3. Overall Study Duration and Follow Up

The study period will consist of screening, treatment, Final Study Visit, and follow-up. The Final Study Visit will occur 28 days after the patient has discontinued study treatment.

8.3.1. Screening

Patient eligibility for the study will be determined within 14 days prior to the first dose of BGB324. Screening assessments will be conducted according to the Schedule of Events tables in Section 10.

8.3.2. Treatment

Eligible patients will visit the study site to receive study treatment and protocol-specified procedures according to the relevant Schedule of Events table in Section 10. The treatment period will consist of continuous 21-day treatment cycles.

Patients will be intensively monitored throughout Day 1 to Day 4 inclusive of Cycle 1, and in addition will attend the clinic on Day 8, and Day 15. Patients will attend the clinic once per week during Cycle 2 and then once per cycle thereafter. All patients will continue to receive BGB324 for as long as, in the opinion of the investigator, they continue to derive clinical benefit or until unacceptable toxicity, disease progression, death or withdrawal of consent.

Patients with AML who are receiving BGB324 monotherapy and who do not achieve an objective response after two cycles of treatment may start additional therapy with oral anti-metabolites (e.g. 6-mercaptopurine, hydroxyurea), or low dose Ara-C, or melphalan administered at 50% of the institution's starting dose, which may be escalated in subsequent cycles according to tolerability.

8.3.3. Final Study Visit

Patients will return to the study site for the Final Study Visit 28 days after discontinuation of BGB324 treatment.

8.3.4. Follow Up

An AE will be monitored for 28 days after the last dose of BGB324. If BGB324-related toxicities continue beyond the follow up period, patients will be followed until all BGB324-related toxicities have resolved, stabilised or returned to baseline. Follow up monitoring for AEs may be conducted over the telephone.

8.4. Study Stopping Rules

BerGenBio AS reserves the right to close a site or terminate the study at any time. The investigator may also terminate the study at their site at any time, provided there is reasonable cause and sufficient notice is given in advance of the intended termination.

Advance notice is not required by either party if the study is stopped due to safety concerns. The reason for closure of an investigational site or termination of a study by BerGenBio AS may include but is not limited to:

- The discovery of an unexpected, serious or unacceptable risk to patients enrolled in the study
- Failure of the investigator to comply with the protocol and Good Clinical Practice (GCP) guidelines
- Inadequate recruitment of patients
- Attainment of the study objectives
- As a result of commercial considerations including discontinuation of further clinical development of BGB324

8.5. End of Study

Except in the case of early termination (Section 8.4), the end of the study is defined as the date when the last patient has completed their last assessment visit.

9. Selection Of Subjects

9.1. Inclusion Criteria

1. Provision of signed written informed consent
2. Histological or cytological confirmation of one of the following:
   a) AML which is sensitive to BGB324 *ex vivo* as defined by an IC_{50} of $\leq$2.5 μM
   b) Part A: High risk group MDS, according to IPSS Risk Stratification, expressing Axl by FCM
   c) Part B: High/intermediate (int-2) risk group MDS, according to IPSS Risk Stratification, expressing Axl by FCM
3. Patients with AML:
   a) Relapsed or refractory disease (Part A)
   b) Newly diagnosed who are unsuitable for intensive chemotherapy (Part B)
4. European Cooperative Oncology Group (ECOG) performance status 0, 1 or 2 [Appendix 1]
5. Male or female, age 18 years or older
6. Female patients of childbearing potential must have a negative serum pregnancy test within 3 days prior to taking their first dose of BGB324. Male patients and female patients of reproductive potential must agree to practice highly effective methods of contraception (such as hormonal implants, combined oral contraceptives, injectable contraceptives, intrauterine device with hormone spirals, total sexual abstinence, vasectomy) throughout the study and for $\geq$3 months after the last dose of BGB324. Female patients are considered NOT of childbearing potential if they have a history of surgical sterility or evidence of post-menopausal status defined as any of the following:
   a) Natural menopause with last menses >1 year ago
   b) Radiation induced oophorectomy with last menses >1 year ago
   c) Chemotherapy induced menopause with last menses >1 year ago

9.2. Exclusion Criteria

1. Pregnant or lactating
2. Abnormal left ventricular ejection fraction on echocardiography (less than the lower limit of normal for a patient of that age at the treating institution)

3. History of an ischaemic cardiac event including myocardial infarction within 3 months of study entry
4. Congestive cardiac failure of > Grade 2 severity according to the NYHA [Appendix 2] as defined as symptomatic at less than ordinary levels of activity
5. Unstable cardiac disease including unstable angina or hypertension as defined by the need for change in medication within the last three months
6. History or presence of bradycardia (less than or equal to 60 BPM) or history of symptomatic bradycardia, left bundle branch block, cardiac pacemaker or significant atrial tachyarrythmias
7. Current treatment with any agent that may prolong QT interval and may cause Torsade de Points [Appendix 3] which cannot be discontinued at least two weeks prior to treatment
8. Known family or personal history of long QTc syndrome or ventricular arrhythmias including ventricular bigeminy
9. Previous history of drug-induced QTc prolongation
10. Screening 12-lead ECG with a measurable QTc interval according to Fridericias correction > 450 ms
11. Abnormal serum potassium, calcium or magnesium levels according to the local laboratory
12. Ongoing infection requiring systemic treatment
13. Inadequate liver function as demonstrated by serum bilirubin ≥1.5 times the upper limits of normal range (ULN) or alanine aminotransferase (ALT) or aspartate aminotransferase (AST) ≥2.5 times the ULN (or ≥5 times the ULN in the presence of liver involvement by leukemia)
14. Inability to tolerate oral medication
15. Existing gastrointestinal disease affecting drug absorption such as celiac disease or Crohns disease
16. Previous bowel resection
17. Evidence of ongoing gastrointestinal graft versus host disease
18. Haematopoetic stem cell transplantation within 6 months
19. Impaired renal function as demonstrated by creatinine ≥1.5 times the ULN or creatinine clearance of ≤50 mL/min determined by Cockcroft-Gault formula
20. Radiotherapy or chemotherapy within the 14 days prior to the first dose of BGB324 being administered (other than hydroxyurea)
21. Receiving an investigational anti-cancer treatment concurrently or within 14 days or five half-lives of either the parent drug or any active metabolite prior to the start of BGB324

22. Unresolved CTCAE Grade 2 or greater toxicity (other than stable toxicity) from previous anti-cancer therapy excluding alopecia
23. Any evidence of severe or uncontrolled systemic conditions (e.g., severe hepatic impairment) or current unstable or uncompensated respiratory or cardiac conditions which makes it undesirable for the patient to participate in the study or which could jeopardize compliance with the protocol
24. Active, uncontrolled central nervous system (CNS) disease including CNS leukemia
25. Active infection with human immunodeficiency virus (HIV), hepatitis B or C viruses – screening for viral infections is **not** required for entry to this study
26. Major surgery within 28 days prior to the start of BGB324 – excluding skin biopsies and procedures for insertion of central venous access devices
27. Known hypersensitivity to cytarabine (Ara-C) or its excipients

10. Study Procedures and Assessments

The Schedule of Events for specific parts of the study are summarised in the following tables as outlined below:

- Part A in Table 3
- Parts B1 and B2 in Table 4
- Part B3 in Table 5

Table 3: Part A Schedule of Events

Cycle		1						2			≥3	End of Study
Week		1				2	3	4	5	6	>7	
Visit	Screen	1	2	3	4	5	6	7	8	9	≥10	
Cycle Day	-14 to 0	1	2	3	4	8	15	1	8	15	1	28 days after last dose
Visit window (+/-) days		0	0	0	0	0	0	2	1	1	2	1
Informed consent	X											
Demographics	X											
Medical history	X											
Inclusion/exclusion	X											
ECOG	X							X			X	X
Physical examination[a]	X	X				X	X	X			X	X
Vital signs[b]	X	X	X	X	X	X	X	X	X	X	X	X
12-lead ECG[c]	X	X	X	X	X	X	X	X	X	X	X	X
Serum pregnancy test[d]	X	X[e]									X[f]	
Clinical chemistry[g]	X	X[e]	X	X	X	X	X	X	X	X	X	X
Hematology[h]	X	X[e]	X	X	X	X	X	X	X	X	X	X
Coagulation screen[i]	X	X[e]				X	X	X			X	X
Urinalysis	X	X[e]				X	X	X	X	X	X	X

Cycle		1						2			≥3	End of Study
Week		1				2	3	4	5	6	>7	
Visit	Screen	1	2	3	4	5	6	7	8	9	≥10	
Cycle Day	-14 to 0	1	2	3	4	8	15	1	8	15	1	28 days after last dose
Bone marrow aspirate	X[j]							X[k]			X[k]	
Echocardiogram	X										X[l]	
PK blood sampling[m]		X	X	X	X	X	X	X	X	X	X	X
PD blood sampling[n]		X	X	X	X	X	X	X[k]	X	X	X[k]	X
Skin/hair PD sample[o]		X										
Hair follicle sampling[p]		X						X				
Response criteria[q]	X							X			X	
Hospital admission[r]		X										
Inpatient hospitalisation[r]			X	X								
Hospital discharge[r]					X							
BGB324 administration[s]		X	X	X	X	X	X	X	X	X	X	
BGB324 accountability						X	X	X	X	X	X	X
Adverse events		X	X	X	X	X	X	X	X	X	X	X
Concomitant medication	X	X	X	X	X	X	X	X	X	X	X	X

a. A full physical examination, excluding gynaecological and rectal examinations, will be conducted at the Screening and End of Study visits. A symptom-directed examination will be conducted at selected visits

b. Vital signs (systolic and diastolic blood pressure (BP), pulse, heart rate and temperature) will be taken at every visit
c. Triplicate 12-lead ECGs will be measured, 5 minutes apart, after resting for ≥10 minutes in supine position at every visit. During Cycle 1 Day 1 – Day 4 triplicate ECGs will be taken to coincide with each PK blood sampling time-point i.e: pre-dose and 2, 4, 6 hr post-dose (Day 1); 20, 24 (pre 2nd dose) and 30 hr post-Day 1 dose (Day 2); 48 hr (pre 3rd dose) post-Day 1 dose (Day 3); and pre-dose, 2, 4 and 6 hr post-dose (Day 4). Patients who re-start BGB324 following interruption for QTc prolongation should also have ECGs performed according to this intensive schedule
d. For women of child-bearing potential
e. Unless conducted as part of the screening procedures within 3 days prior to Day 1
f. Cycle 3 and every other Cycle thereafter
g. Clinical chemistry laboratory parameters (uric acid, electrolytes, blood urea nitrogen (BUN), total protein, total bilirubin, alanine aminotransferase (ALT), aspartate aminotransferase (AST), serum creatinine, creatine phosphokinase, alkaline phosphatase, albumin, calcium, phosphorus, glucose)
h. Hematology laboratory parameters (full blood count including differential white cell count, haemoglobin, haematocrit and platelets)
i. Coagulation parameters: prothrombin time (PT) and activated partial thromboplastin time (APPT)
j. Bone marrow aspirate for diagnosis, eligibility (sensitivity to BGB324 in ex vivo assay (AML) & axl expression (MDS)) and baseline sample for PD biomarker analyses
k. Bone marrow aspirate (pre-dose) for clinical response assessment and PD biomarker analyses. If BM aspirate sample volume is insufficient a blood sample will be taken for PD biomarker analyses
l. Echocardiogram Cycle 4 Day 1 ONLY
m. Blood sampling for the determination of BGB324 in plasma will be conducted at the following Cycle 1 time-points: pre-dose and 2, 4, 6 hr post-dose (Day 1); 20, 24 (pre 2nd dose) and 30 hr post-Day 1 dose (Day 2); 48 hr (pre 3rd dose) post-Day 1 dose (Day 3); and pre-dose, 2, 4 and 6 hr post-dose (Day 4). A window of +/- 1 hr and +/- 2 hr applies to the Cycle 1 Day 1 6 hr and 20 hr post-dose time-points respectively. A pre-dose sample will be collected at ALL study visits (including ≥3 Cycle 3 and End of Study)
n. Blood sampling for assessment of BGB324 activity by PD biomarker analyses will be conducted at the following Cycle 1 time-points: pre-dose and 2, 4, 6 hr post-dose (Day 1); 20, 24 (pre 2nd dose) and 30 hr post-Day 1 dose (Day 2); 48 hr (pre 3rd dose) post-Day 1 dose (Day 3); and pre-dose, 2, 4 and 6 hr post-dose (Day 4). A window of +/- 1 hr and +/- 2 hr applies to the Cycle 1 Day 1 6 hr and 20 hr post-dose time-points respectively. A pre-dose sample will be collected at ALL study visits (including ≥3 Cycle 3 and End of Study)
o. Skin biopsy or hair follicles (according to local practice) for PD biomarker genomics analysis (pre-dose Cycle 1 Day 1 ONLY)
p. Hair follicle sampling for assessment of BGB324 activity by PD biomarker analyses will be conducted pre-dose on Day 1 of Cycle 1 and Cycle 2
q. Assessment of clinical response (% blasts; neutrophil count; platelet count (& haemoglobin (MDS ONLY))
r. Optional in-patient hospitalisation: for convenience patients can be admitted for in-patient stay on Day 1 and discharged on Day 4
s. BGB324 should be taken, with food, at approximately the same time on each dosing day and patients should record dosing in the dosing diary provided. On study visit days, BGB324 should be administered on the unit

Table 4: Parts B1 and B2 Schedule of Events

Cycle		1						2			≥3	End of Study
Week		1				2	3	4	5	6	>7	
Visit	Screen	1	2	3	4	5	6	7	8	9	≥10	
Cycle Day	-14 to 0	1	2	3	4	8	15	1	8	15	1	28 days after last dose
Visit window (+/-) days		0	0	0	0	0	0	2	1	1	2	1
Informed consent	X											
Demographics	X											
Medical history	X											
Inclusion/exclusion	X											
ECOG	X							X			X	X
Physical examination[a]	X	X				X	X	X			X	X
Vital signs[b]	X	X	X	X	X	X	X	X	X	X	X	X
12-lead ECG[c]	X	X	X	X	X	X	X	X	X	X	X	X
Serum pregnancy test[d]	X	X[e]									X[f]	
Clinical chemistry[g]	X	X[e]	X	X	X	X	X	X	X	X	X	X
Hematology[h]	X	X[e]	X	X	X	X	X	X	X	X	X	X
Coagulation screen[i]	X	X[e]				X	X	X			X	X
Urinalysis	X	X[e]				X	X	X	X	X	X	X

Cycle		1						2			≥3	End of Study
Week		1				2	3	4	5	6	>7	
Visit	Screen	1	2	3	4	5	6	7	8	9	≥10	
Cycle Day	-14 to 0	1	2	3	4	8	15	1	8	15	1	28 days after last dose
Bone marrow aspirate	X[j]							X[k]			X[k]	
Echocardiogram	X										X[l]	
PD blood sampling[m]		X	X	X	X	X	X	X[k]	X	X	X[k]	X
Skin/hair PD sample[n]		X										
Hair follicle sampling[o]		X						X				
Response criteria[p]	X							X			X	
Hospital admission[q]		X										
Inpatient hospitalisation[q]			X	X								
Hospital discharge[q]					X							
BGB324 administration[r]		X	X	X	X	X	X	X	X	X	X	
BGB324 accountability						X	X	X	X	X	X	X
Adverse events		X	X	X	X	X	X	X	X	X	X	X
Concomitant medication	X	X	X	X	X	X	X	X	X	X	X	X

a. A full physical examination, excluding gynaecological and rectal examinations, will be conducted at the Screening and End of Study visits. A symptom-directed examination will be conducted at selected visits
b. Vital signs (systolic and diastolic blood pressure (BP), pulse, heart rate and temperature) will be taken at every visit

c. Triplicate 12-lead ECGs will be measured, 5 minutes apart, after resting for $\geq$10 minutes in supine position at every visit. During Cycle 1 Day 1 – Day 4 triplicate ECGs will be taken at the following time-points: pre-dose and 2, 4, 6 hr post-dose (Day 1); 20, 24 (pre 2nd dose) and 30 hr post-Day 1 dose (Day 2); 48 hr (pre 3rd dose) post-Day 1 dose (Day 3); and pre-dose, 2, 4 and 6 hr post-dose (Day 4). Patients who re-start BGB324 following interruption for QTc prolongation should also have ECGs performed according to this intensive schedule

d. For women of child-bearing potential

e. Unless conducted as part of the screening procedures within 3 days prior to Day 1

f. Cycle 3 and every other Cycle thereafter

g. Clinical chemistry laboratory parameters (uric acid, electrolytes, blood urea nitrogen (BUN), total protein, total bilirubin, alanine aminotransferase (ALT), aspartate aminotransferase (AST), serum creatinine, creatine phosphokinase, alkaline phosphatase, albumin, calcium, phosphorus, glucose)

h. Hematology laboratory parameters (full blood count including differential white cell count, haemoglobin, haematocrit and platelets)

i. Coagulation parameters: prothrombin time (PT) and activated partial thromboplastin time (APPT)

j. Bone marrow aspirate for diagnosis, eligibility (sensitivity to BGB324 in ex vivo assay (AML) & axl expression (MDS)) and baseline sample for PD biomarker analyses

k. Bone marrow aspirate (pre-dose) for clinical response assessment and PD biomarker analyses. If BM aspirate sample volume is insufficient a blood sample will be taken for PD biomarker analyses

l. Echocardiogram Cycle 4 Day 1 ONLY

m. Blood sampling for assessment of BGB324 activity by PD biomarker analyses will be conducted at the following Cycle 1 time-points: pre-dose (Day 1); 24hr (pre 2nd dose) post-Day 1 dose (Day 2); 48 hr (pre 3rd dose) post-Day 1 dose (Day 3); and pre-dose (Day 4). A pre-dose sample will be collected at ALL study visits (including $\geq$3 Cycle 3 and End of Study)

n. Skin biopsy or hair follicles (according to local practice) for PD biomarker genomics analysis (pre-dose Cycle 1 Day 1 ONLY)

o. Hair follicle sampling for assessment of BGB324 activity by PD biomarker analyses will be conducted pre-dose on Day 1 of Cycle 1 and Cycle 2

p. Assessment of clinical response (% blasts; neutrophil count; platelet count (& haemoglobin MDS ONLY))

q. Optional in-patient hospitalisation: for convenience patients can be admitted for in-patient stay on Day 1 and discharged on Day 4

r. BGB324 should be taken, with food, at approximately the same time on each dosing day and patients should record dosing in the dosing diary provided. On study visit days, BGB324 should be administered on the unit

Table 5: Part B3 Schedule of Events

**Cycle**		**1**						**2**			**≥3**	**End of Study**
**Week**		**1**				**2**	**3**	**4**	**5**	**6**	**>7**	
**Visit**	**Screen**	**1**	**2**	**3**	**4**	**5**	**6**	**7**	**8**	**9**	**≥10**	
**Cycle Day**	**-14 to 0**	**1**	**2**	**3**	**4**	**8**	**15**	**1**	**8**	**15**	**1**	**28 days after last dose**
Visit window (+/-) days		0	0	0	0	0	0	2	1	1	2	1
Informed consent	X											
Demographics	X											
Medical history	X											
Inclusion/exclusion	X											
ECOG	X							X			X	X
Physical examination[a]	X	X				X	X	X			X	X
Vital signs[b]	X	X	X	X	X	X	X	X	X	X	X	X
12-lead ECG[c]	X	X	X	X	X	X	X	X	X	X	X	X
Serum pregnancy test[d]	X	X[e]									X[f]	
Clinical chemistry[g]	X	X[e]	X	X	X	X	X	X	X	X	X	X
Hematology[h]	X	X[e]	X	X	X	X	X	X	X	X	X	X
Coagulation screen[i]	X	X[e]				X	X	X			X	X
Urinalysis	X	X[e]				X	X	X	X	X	X	X

Cycle		1						2			≥3	End of Study
Week		1				2	3	4	5	6	>7	
Visit	Screen	1	2	3	4	5	6	7	8	9	≥10	
Cycle Day	-14 to 0	1	2	3	4	8	15	1	8	15	1	28 days after last dose
Bone marrow aspirate	X[j]							X[k]			X[k]	
Echocardiogram	X										X[l]	
PK blood sampling[m]		X	X	X	X	X	X	X	X	X	X	X
PK blood sampling[n]		X										
PD blood sampling[o]		X	X	X	X	X	X	X[k]	X	X	X[k]	X
Skin/hair PD sample[p]		X										
Hair follicle sampling[q]		X						X				
Response criteria[r]	X							X			X	
Hospital admission[s]		X										
Inpatient hospitalisation[s]			X	X								
Hospital discharge[s]					X							
BGB324 administration[t]		X	X	X	X	X	X	X	X	X	X	
Ara-C administration[u]		X	X	X	X	X		(X)	(X)	(X)	(X)	
BGB324 accountability						X	X	X	X	X	X	X
Adverse events		X	X	X	X	X	X	X	X	X	X	X

Cycle		1						2			≥3	End of Study
Week		1				2	3	4	5	6	>7	
Visit	Screen	1	2	3	4	5	6	7	8	9	≥10	
Cycle Day	-14 to 0	1	2	3	4	8	15	1	8	15	1	28 days after last dose
Concomitant medication	X	X	X	X	X	X	X	X	X	X	X	X

a. A full physical examination, excluding gynaecological and rectal examinations, will be conducted at the Screening and End of Study visits. A symptom-directed examination will be conducted at selected visits
b. Vital signs (systolic and diastolic blood pressure (BP), pulse, heart rate and temperature) will be taken at every visit
c. Triplicate 12-lead ECGs will be measured, 5 minutes apart, after resting for ≥10 minutes in supine position at every visit. During Cycle 1 Day 1 – Day 4 triplicate ECGs will be taken to coincide with each PK blood sampling time-point i.e: pre-dose and 2, 4, 6 hr post-dose (Day 1); 20, 24 (pre 2nd dose) and 30 hr post-Day 1 dose (Day 2); 48 hr (pre 3rd dose) post-Day 1 dose (Day 3); and pre-dose, 2, 4 and 6 hr post-dose (Day 4). Patients who re-start BGB324 following interruption for QTc prolongation should also have ECGs performed according to this intensive schedule
d. For women of child-bearing potential
e. Unless conducted as part of the screening procedures within 3 days prior to Day 1
f. Cycle 3 and every other Cycle thereafter
g. Clinical chemistry laboratory parameters (uric acid, electrolytes, blood urea nitrogen (BUN), total protein, total bilirubin, alanine aminotransferase (ALT), aspartate aminotransferase (AST), serum creatinine, creatine phosphokinase, alkaline phosphatase, albumin, calcium, phosphorus, glucose)
h. Hematology laboratory parameters (full blood count including differential white cell count, haemoglobin, haematocrit and platelets)
i. Coagulation parameters: prothrombin time (PT) and activated partial thromboplastin time (APPT)
j. Bone marrow aspirate for diagnosis, eligibility (sensitivity to BGB324 in ex vivo assay (AML) & axl expression (MDS)) and baseline sample for PD biomarker analyses
k. Bone marrow aspirate (pre-dose) for clinical response assessment and PD biomarker analyses. If BM aspirate sample volume is insufficient a blood sample will be taken for PD biomarker analyses
l. Echocardiogram Cycle 4 Day 1 ONLY
m. Blood sampling for the determination of BGB324 in plasma will be conducted at the following Cycle 1 time-points: pre-dose and 2, 4, 6 hr post-dose (Day 1); 20, 24 (pre 2nd dose) and 30 hr post-Day 1 dose (Day 2); 48 hr (pre 3rd dose) post-Day 1 dose (Day 3); and pre-dose, 2, 4 and 6 hr post-dose (Day 4). A window of +/- 1 hr and +/- 2 hr applies to the Cycle 1 Day 1 6 hr and 20 hr post-dose time-points respectively. A pre-dose sample will be collected at ALL study visits (including ≥3 Cycle 3 and End of Study)
n. Blood sampling for the determination of Ara-C in plasma will be conducted at the following Cycle 1 time-points: pre-dose and 0.25, 0.5, 1.5, 2, 4, 6 hr post-dose (Day 1)
o. Blood sampling for assessment of BGB324 activity by PD biomarker analyses will be conducted at the following Cycle 1 time-points: pre-dose and 2, 4, 6 hr post-dose (Day 1); 20, 24 (pre 2nd dose) and 30 hr post-Day 1 dose (Day 2); 48 hr (pre 3rd dose) post-Day 1 dose (Day 3); and pre-dose, 2, 4 and 6 hr post-dose (Day 4). A

window of +/- 1 hr and +/- 2 hr applies to the Cycle 1 Day 1 6 hr and 20 hr post-dose time-points respectively. A pre-dose sample will be collected at ALL study visits (including ≥3 Cycle 3 and End of Study)

p. Skin biopsy or hair follicles (according to local practice) for PD biomarker genomics analysis (pre-dose Cycle 1 Day 1 ONLY)

q. Hair follicle sampling for assessment of BGB324 activity by PD biomarker analyses will be conducted pre-dose on Day 1 of Cycle 1 and Cycle 2

r. Assessment of clinical response (% blasts; neutrophil count; platelet count & haemoglobin (MDS ONLY))

s. Optional in-patient hospitalisation: for convenience patients can be admitted for in-patient stay on Day 1 and discharged on Day 4

t. BGB324 should be taken, with food, at approximately the same time on each dosing day and patients should record dosing in the dosing diary provided. On study visit days, BGB324 should be administered on the unit

u. Ara-C will be administered at a dose of $10mg/m^2$ administered subcutaneously twice daily for up to fourteen consecutive days repeated every four to six weeks depending upon the patient's haematological recovery

10.1. Enrolment

Patients must provide written informed consent before any screening procedures are performed. Patients who meet all inclusion/exclusion criteria will be allocated a study-specific patient number.

In Part A the order of entry into the study will determine a patient's cohort and corresponding dose level. Patients will be assigned the next available ascending patient number. In Part B patients will be assigned to a specific cohort (B1, B2 or B3) dependent on their hematological malignancy. Patients will be assigned the next available ascending patient number.

10.1.1. Blinding Procedures

This is an open-label study and consequently there are no blinding procedures in operation.

10.2. Screening

During screening, the following procedures will be performed:

- Obtain written informed consent
- Demographic data, including date of birth, sex, height, and race
- Medical and surgical history
- Concomitant medication
- Physical examination, including weight
- Echocardiogram
- Vital sign measurements (systolic and diastolic BP, pulse, heart rate and temperature)
- Serial 12-lead ECGs, in triplicate (≥5 minutes apart), performed after the patient has been supine for 5 minutes)
- ECOG performance status [Appendix 1]
- Collection of blood sample for hematology (including neutrophil and platelet count), clinical chemistry profile
- Coagulation panel
- Routine urinalysis
- Serum pregnancy test, where appropriate
- BM aspirate for confirmation of the diagnosis and for ex vivo BGB324 sensitivity assay (AML) or Axl surface expression (MDS) for confirmation of eligibility and for PD biomarker analyses

Male patients and female patients of reproductive potential must agree to practice highly effective methods of contraception throughout the study and for $\geq$3 months after the last dose of BGB324. Highly effective methods of contraception are defined as:

- Hormonal implants, combined oral contraceptives, injectable contraceptives,
- An intrauterine device with hormone spirals
- True total sexual abstinence
- Vasectomy

If it is not possible to use one of these highly effective methods of contraception two barrier methods used in conjunction are acceptable.

Patients should be advised to avoid exposing their skin to direct sunlight throughout study participation by practicing the following effective methods:

- Wearing clothing to protect exposed areas of the skin including the head, face, arms and legs
- Applying lip balm and a broad spectrum sunscreen (minimum of Sun Protection Factor (SPF) 30) to exposed areas of the skin every 2 to 3 hours

10.3. Treatment Period

The following sections outline the assessments to be conducted in each part of the study. For Cycle 2 and beyond a visit window of +/-48 hours is allowed for the Day 1 visit and +/-24 hours for other visits, per protocol.

10.3.1. Part A

Please refer to the Schedule of Events outlined in Table 3.

10.3.1.1. Cycle 1

In addition to the assessments below, AEs and concomitant medications will be recorded on each visit. Patients will be required to attend the clinic for each dosing day during Cycle 1. Consequently, and if it is more convenient, patients can be admitted for in-patient hospitalisation on Day 1 and discharged on Day 4.

Visit 1 (Day 1):

- Hospital admission (if applicable)
- Physical examination, including weight
- Collection of blood sample for hematology (including neutrophil and platelet count) and clinical chemistry profile (unless conducted as part of the screening procedures within 3 days prior to Day 1)
- Coagulation panel (unless conducted as part of the screening procedures within 3 days prior to Day 1)

- Routine urinalysis unless conducted as part of the screening procedures within 3 days prior to Day 1)
- 12-lead ECGs per screening procedures (pre-dose and 2, 4, and 6 hr post-dose)
- Vital signs per screening procedures
- Collection of PK blood samples (at time-points outlined in Table 3)
- Collection of PD biomarker blood samples (at time-points outlined in Table 3)
- Collection of PD genomics biomarker skin biopsy or hair follicles according to local practice (pre-dose)
- Collection of PD biomarker hair follicle sample (pre-dose)
- BGB324 administration

Visit 2 (Day 2):

- In-patient stay (if applicable)
- Collection of blood sample for hematology (including neutrophil and platelet count) and clinical chemistry profile
- 12-lead ECGs per screening procedures (20, 24 and 30 hr post Day 1 dose)
- Vital signs per screening procedures
- Collection of PK blood samples (at time-points outlined in Table 3)
- Collection of PD biomarker blood samples (at time-points outlined in Table 3)
- BGB324 administration

Visit 3 (Day 3):

- In-patient stay (if applicable)
- Collection of blood sample for hematology (including neutrophil and platelet count) and clinical chemistry profile
- 12-lead ECGs per screening procedures (48 hr post Day 1 dose)
- Vital signs per screening procedures
- Collection of PK blood samples (at time-points outlined in Table 3)
- Collection of PD biomarker blood samples (at time-points outlined in Table 3)
- BGB324 administration

Visit 4 (Day 4):

- Hospital discharge (if applicable)
- Collection of blood sample for hematology (including neutrophil and platelet count) and clinical chemistry profile
- 12-lead ECGs per screening procedures (pre-dose and 2, 4, and 6 hr post-dose)
- Vital signs per screening procedures
- Collection of PK blood samples (at time-points outlined in Table 3)

- Collection of PD biomarker blood samples (at time-points outlined in Table 3)
- BGB324 administration

Visit 5 (Day 8):

- Physical examination, including weight
- Collection of blood sample for hematology (including neutrophil and platelet count) and clinical chemistry profile
- Coagulation panel
- 12-lead ECGs per screening procedures
- Vital signs per screening procedures
- Routine urinalysis
- Collection of PK blood sample (pre-dose)
- Collection of PD biomarker blood sample (pre-dose)
- BGB324 administration
- BGB324 accountability

Visit 6 (Day 15):

- Physical examination, including weight
- Collection of blood sample for hematology (including neutrophil and platelet count) and clinical chemistry profile
- Coagulation panel
- 12-lead ECGs per screening procedures
- Vital signs per screening procedures
- Collection of PK blood samples (pre-dose)
- Collection of PD biomarker blood samples (pre-dose)
- Routine urinalysis
- BGB324 administration
- BGB324 accountability

10.3.2. Cycle 2

In addition to the assessments below, AEs and concomitant medications will be recorded on each visit.

Visit 7 (Day 1):

- Physical examination, including weight
- ECOG performance status [Appendix 1]
- Collection of blood sample for hematology (including neutrophil and platelet count) and clinical chemistry profile

- Coagulation panel
- Routine urinalysis
- 12-lead ECGs per screening procedures
- Vital signs per screening procedures
- Collection of PK blood sample (pre-dose)
- Collection of PD biomarker blood sample (pre-dose)
- Collection of PD biomarker hair follicle sample (pre-dose)
- BGB324 administration
- BGB324 accountability
- BM aspirate for clinical response criteria review and PD biomarker analyses (pre-dose)
- Collection of PD biomarker blood sample (pre-dose) ONLY if BM aspirate sample volume is insufficient
- Clinical response criteria review

Visit 8 (Day 8) and Visit 9 (Day 15):

- Collection of blood sample for hematology (including neutrophil and platelet count) and clinical chemistry profile
- Routine urinalysis
- 12-lead ECGs per screening procedures
- Vital signs per screening procedures
- Collection of PK blood sample (pre-dose)
- Collection of PD biomarker blood sample (pre-dose)
- BGB324 administration
- BGB324 accountability

10.3.3. Cycle 3 and Subsequent Cycles

In addition to the assessments below, AEs and concomitant medications will be recorded on each visit.

BM aspirates, ECGs and laboratory investigations should be taken more frequently if clinically indicated.

Visit $\geq$10 (Day 1):

- Serum pregnancy test for women of childbearing potential (Cycle 3 and every other Cycle thereafter)
- Physical examination, including weight
- ECOG performance status [Appendix 1]
- Echocardiogram (Cycle 4 ONLY)

- Collection of blood sample for hematology (including neutrophil and platelet count for clinical response criteria review) and clinical chemistry profile
- Coagulation panel
- Routine urinalysis
- 12-lead ECGs per screening procedures
- Vital signs per screening procedures
- Collection of PK blood sample (pre-dose)
- Collection of PD biomarker blood sample (pre-dose)
- BM aspirate for clinical response criteria review and PD biomarker analyses (pre-dose)
- Clinical response criteria review
- Collection of PD biomarker blood sample (pre-dose) ONLY if BM aspirate sample volume is insufficient
- BGB324 administration
- BGB324 accountability

10.3.4. Part B1 and Part B2

Please refer to the Schedule of Events outlined in Table 4.

10.3.4.1. Cycle 1

In addition to the assessments below, AEs and concomitant medications will be recorded on each visit. Patients will be required to attend the clinic for each dosing day during Cycle 1. Consequently, and if it is more convenient, patients can will be admitted for in-patient hospitalisation on Day 1 and discharged on Day 4.

Visit 1 (Day 1):

- Hospital admission (if applicable)
- Physical examination, including weight
- Collection of blood sample for hematology (including neutrophil and platelet count) and clinical chemistry profile (unless conducted as part of the screening procedures within 3 days prior to Day 1)
- Coagulation panel (unless conducted as part of the screening procedures within 3 days prior to Day 1)
- Routine urinalysis unless conducted as part of the screening procedures within 3 days prior to Day 1)
- 12-lead ECGs per screening procedures (pre-dose and 2, 4, and 6 hr post-dose)
- Vital signs per screening procedures
- Collection of PD biomarker blood sample (pre-dose)

- Collection of PD genomics biomarker skin biopsy or hair follicles according to local practice (pre-dose)
- Collection of PD biomarker hair follicle sample (pre-dose)
- BGB324 administration

Visit 2 (Day 2):

- In-patient stay (if applicable)
- Collection of blood sample for hematology (including neutrophil and platelet count) and clinical chemistry profile
- 12-lead ECGs per screening procedures (20, 24 and 30 hr post Day 1 dose)
- Vital signs per screening procedures
- Collection of PD biomarker blood samples (at time-points outlined in Table 4)
- BGB324 administration

Visit 3 (Day 3):

- In-patient stay (if applicable)
- Collection of blood sample for hematology (including neutrophil and platelet count) and clinical chemistry profile
- 12-lead ECGs per screening procedures (48 hr post Day 1 dose)
- Vital signs per screening procedures
- BGB324 administration

Visit 4 (Day 4):

- Hospital discharge (if applicable)
- Collection of blood sample for hematology (including neutrophil and platelet count) and clinical chemistry profile
- 12-lead ECGs per screening procedures (pre-dose and 2, 4, and 6 hr post-dose)
- Vital signs per screening procedures
- BGB324 administration

Visit 5 (Day 8):

- Physical examination, including weight
- Collection of blood sample for hematology (including neutrophil and platelet count) and clinical chemistry profile
- Coagulation panel
- 12-lead ECGs per screening procedures
- Vital signs per screening procedures
- Routine urinalysis

- BGB324 administration
- BGB324 accountability

Visit 6 (Day 15):

- Physical examination, including weight
- Collection of blood sample for hematology (including neutrophil and platelet count) and clinical chemistry profile
- Coagulation panel
- 12-lead ECGs per screening procedures
- Vital signs per screening procedures
- Collection of PD biomarker blood sample (pre-dose)
- Routine urinalysis
- BGB324 administration
- BGB324 accountability

10.3.5. Cycle 2

In addition to the assessments below, AEs and concomitant medications will be recorded on each visit.

Visit 7 (Day 1):

- Physical examination, including weight
- ECOG performance status [Appendix 1]
- Collection of blood sample for hematology (including neutrophil and platelet count) and clinical chemistry profile
- Coagulation panel
- Routine urinalysis
- 12-lead ECGs per screening procedures
- Vital signs per screening procedures
- Collection of PD biomarker blood samples (pre-dose)
- BGB324 administration
- BGB324 accountability
- BM aspirate for clinical response criteria review and PD biomarker analyses (pre-dose)
- Clinical response criteria review
- Collection of PD biomarker blood sample (pre-dose) ONLY if BM aspirate sample volume is insufficient

Visit 8 (Day 8) and Visit 9 (Day 15):

- Physical examination, including weight
- Collection of blood sample for hematology (including neutrophil and platelet count) and clinical chemistry profile
- Routine urinalysis
- 12-lead ECGs per screening procedures
- Vital signs per screening procedures
- BGB324 administration
- BGB324 accountability

10.3.6. Cycle 3 and Subsequent Cycles

In addition to the assessments below, AEs and concomitant medications will be recorded on each visit.

BM aspirates should be taken more frequently if clinically indicated.

Visit $\geq$10 (Day 1):

- Serum pregnancy test for women of childbearing potential (Cycle 3 and every other Cycle thereafter)
- Physical examination, including weight
- ECOG performance status [Appendix 1]
- Echocardiogram (Cycle 4 ONLY)
- Collection of blood sample for hematology (including neutrophil and platelet count for clinical response criteria review) and clinical chemistry profile
- Coagulation panel
- Routine urinalysis
- 12-lead ECGs per screening procedures
- Vital signs per screening procedures
- Collection of PD biomarker blood samples (pre-dose)
- BM aspirate for clinical response criteria review and PD biomarker analyses (pre-dose)
- Clinical response criteria review
- Collection of PD biomarker blood sample (pre-dose) ONLY if BM aspirate sample volume is insufficient
- BGB324 administration
- BGB324 accountability

10.3.7. Part B3

Please refer to the Schedule of Events outlined in Table 5. Ara-C will be administered at a dose of 10mg/m^2 administered subcutaneously twice daily for up to fourteen consecutive

days repeated every four to six weeks depending upon the patient's haematological recovery.

10.3.7.1. Cycle 1

In addition to the assessments below, AEs and concomitant medications will be recorded on each visit. Patients will be required to attend the clinic for each dosing day during Cycle 1. Consequently, and if it is more convenient, patients can will be admitted for in-patient hospitalisation on Day 1 and discharged on Day 4.

Visit 1 (Day 1):

- Hospital admission (if applicable)
- Physical examination, including weight
- Collection of blood sample for hematology (including neutrophil and platelet count) and clinical chemistry profile (unless conducted as part of the screening procedures within 3 days prior to Day 1)
- Coagulation panel (unless conducted as part of the screening procedures within 3 days prior to Day 1)
- Routine urinalysis unless conducted as part of the screening procedures within 3 days prior to Day 1)
- 12-lead ECGs per screening procedures (pre-dose and 2, 4, and 6 hr post-dose)
- Vital signs per screening procedures
- Collection of PK blood samples for BGB324 and Ara-C analysis (at time-points outlined in Table 5)
- Collection of PD biomarker blood samples (at time-points outlined in Table 5)
- Collection of PD genomics biomarker skin biopsy or hair follicles according to local practice (pre-dose)
- Collection of PD biomarker hair follicle sample (pre-dose)
- BGB324 administration
- Ara-C administration

Visit 2 (Day 2):

- In-patient stay (if applicable)
- Collection of blood sample for hematology (including neutrophil and platelet count) and clinical chemistry profile
- 12-lead ECGs per screening procedures (20, 24, and 30 hr post Day 1 dose)
- Vital signs per screening procedures
- Collection of PK blood samples (at time-points outlined in Table 5)
- Collection of PD biomarker blood samples (at time-points outlined in Table 5)

- BGB324 administration
- Ara-C administration

Visit 3 (Day 3):

- In-patient stay (if applicable)
- Collection of blood sample for hematology (including neutrophil and platelet count) and clinical chemistry profile
- 12-lead ECGs per screening procedures (48 hr post Day 1 dose)
- Vital signs per screening procedures
- Collection of PK blood samples (at time-points outlined in Table 5)
- Collection of PD biomarker blood samples (at time-points outlined in Table 5)
- BGB324 administration
- Ara-C administration

Visit 4 (Day 4):

- Hospital discharge (if applicable)
- Collection of blood sample for hematology (including neutrophil and platelet count) and clinical chemistry profile
- 12-lead ECGs per screening procedures (pre-dose and 2, 4, and 6 hr post-dose)
- Vital signs per screening procedures
- Collection of PK blood samples (at time-points outlined in Table 5)
- Collection of PD biomarker blood samples (at time-points outlined in Table 5)
- Ara-C administration
- BGB324 administration

Visit 5 (Day 8):

- Physical examination, including weight
- Collection of blood sample for hematology (including neutrophil and platelet count) and clinical chemistry profile
- Coagulation panel
- 12-lead ECGs per screening procedures
- Vital signs per screening procedures
- Routine urinalysis
- Collection of PK blood sample (pre-dose)
- Collection of PD biomarker blood sample (pre-dose)
- BGB324 administration
- BGB324 accountability
- Ara-C administration (if applicable)

Visit 6 (Day 15):

- Physical examination, including weight
- Collection of blood sample for hematology (including neutrophil and platelet count) and clinical chemistry profile
- Coagulation panel
- 12-lead ECGs per screening procedures
- Vital signs per screening procedures
- Collection of PK blood samples (pre-dose)
- Collection of PD biomarker blood samples (pre-dose)
- Routine urinalysis
- BGB324 administration
- BGB324 accountability

10.3.8. Cycle 2

In addition to the assessments below, AEs and concomitant medications will be recorded on each visit.

Visit 7 (Day 1):

- Physical examination, including weight
- ECOG performance status [Appendix 1]
- Collection of blood sample for hematology (including neutrophil and platelet count) and clinical chemistry profile
- Coagulation panel
- Routine urinalysis
- 12-lead ECGs per screening procedures
- Vital signs per screening procedures
- Collection of PK blood sample (pre-dose)
- Collection of PD biomarker blood sample (pre-dose)
- Collection of PD biomarker hair follicle sample (pre-dose)
- BGB324 administration
- BGB324 accountability
- BM aspirate for clinical response criteria review and PD biomarker analyses (pre-dose)
- Collection of PD biomarker blood sample (pre-dose) ONLY if BM aspirate sample volume is insufficient
- Clinical response criteria review

Visit 8 (Day 8) and Visit 9 (Day 15):

- Collection of blood sample for hematology (including neutrophil and platelet count) and clinical chemistry profile
- Routine urinalysis
- 12-lead ECGs per screening procedures
- Vital signs per screening procedures
- Collection of PK blood sample (pre-dose)
- Collection of PD biomarker blood sample (pre-dose)
- BGB324 administration
- BGB324 accountability
- Ara-C administration (if applicable)

10.3.9. Cycle 3 and Subsequent Cycles

In addition to the assessments below, AEs and concomitant medications will be recorded on each visit.

BM aspirates should be taken more frequently if clinically indicated.

Visit ≥10 (Day 1):

- Serum pregnancy test for women of childbearing potential (Cycle 3 and every other Cycle thereafter)
- Physical examination, including weight
- ECOG performance status [Appendix 1]
- Echocardiogram (Cycle 4 ONLY)
- Collection of blood sample for hematology (including neutrophil and platelet count for clinical response criteria review) and clinical chemistry profile
- Coagulation panel
- Routine urinalysis
- 12-lead ECGs per screening procedures
- Vital signs per screening procedures
- Collection of PK blood sample (pre-dose)
- Collection of PD biomarker blood sample (pre-dose)
- BM aspirate for clinical response criteria review and PD biomarker analyses (pre-dose)
- Clinical response criteria review
- BGB324 administration
- BGB324 accountability
- Ara-C administration (if applicable)

10.3.10. Final Study Visit

The following assessments will be carried out for every patient enrolled into the study 28 days after the last dose of study medication or at study withdrawal:

- Collection of blood sample for hematology (including neutrophil and platelet count for clinical response criteria review) and clinical chemistry profile
- Coagulation panel
- Routine urinalysis
- Recording of AEs
- Concomitant medications
- Physical examination, including weight
- ECOG performance status [Appendix 1]
- Vital signs per screening procedures
- Clinical response criteria review
- 12-lead ECGs per screening procedures
- Collection of PK blood sample
- Collection of PD biomarker blood sample
- BGB324 accountability

11. Treatment Of Subjects

BerGenBio AS will provide BGB324 to each study site.

Please refer to Section 13 for the procedures for preparation, storage, handling, disposal and accountability of BGB324.

In Part B3 Ara-C will be administered at a dose of 10mg/m^2 administered subcutaneously twice daily for up to fourteen consecutive days repeated every four to six weeks depending upon the patient's haematological recovery.

11.1. Treatment Schedule and Administration

11.1.1. BGB324

In Part A, the starting dose of BGB324 (Cohort 1) will be 400 mg on Cycle 1 Day 1 and Day 2 (loading dose), followed by a daily maintenance dose of 100 mg.

At a minimum BGB324 should be taken following 2 slices of buttered toast and 100 mL of full fat milk to optimise drug absorption.

Patients will record their dosing at home in a dosing diary that will be reviewed regularly by the site staff. Patients will be instructed as to the importance of following the dosing instructions they have been provided with, and for maintaining an accurate and up to date dosing record. Patients will also be instructed to bring their dosing diary and all BGB324

bottles, including unused and empty bottles, with them to each study visit. The site staff will record the amount of used and unused BGB324 capsules at selected study visits.

11.1.2. Ara-C

In Part B3 Ara-C will be administered for up to fourteen consecutive days repeated every four to six weeks depending upon the patient's haematological recovery. Ara-C is considered to be standard of care for these patients and will not be supplied or reimbursed by BerGenBio AS.

11.2. Dose Modification

11.2.1. BGB324

If a patient experiences drug-related toxicity which requires treatment with BGB324 to be interrupted, a delay of up to 14 days is permitted to allow for resolution of toxicity. Toxicities must have resolved to Grade ≤I or baseline for treatment to recommence. For Grade I or Grade 2 (tolerable) toxicity, the patient may resume dosing at the same dose level. For ≥Grade 2 (intolerable) toxicity, the patient may resume dosing at a dose level defined by the grade of the toxicity and the number of occurrence(s) of prior toxicity as outlined in Table 6. A maximum of two dose reductions are permitted.

Table 6: Dose Modification of BGB324 for Toxicity

Grade (CTCAE)	Recommended dose modification
**Grade I or Grade 2 (tolerable)**	Maintain starting dose
**Grade 2 (intolerable) or Grade 3**	
1st occurrence	Interrupt treatment until Grade 0 – I<br>Resume dosing at Reduced Dose Level (-1)
2nd occurrence of any Grade 2 or 3 AE or persistence after treatment interruption	Interrupt treatment until Grade 0 – I<br>Resume dosing at Reduced Dose Level (-2)<br><u>or</u> discontinue permanently if dose has already been lowered to Reduced Dose Level (-2)
3rd occurrence of any Grade 2 or 3 AE or persistence after 2nd dose reduction	Discontinue permanently
**Grade 4**	
1st occurrence	Discontinue permanently or interrupt treatment until Grade 0 – I<br>Resume dosing at Reduced Dose Level (-2)<br><u>or</u> discontinue permanently if dose has already been lowered
2nd occurrence of any Grade 4 AE or persistence of any Grade 4 AE after 1st dose reduction	Discontinue permanently

If after a fourteen-day delay, toxicity has not resolved to Grade ≤I or baseline the patient should be withdrawn from the study.

In clinical study BGBC001 BGB324 exhibited evidence of exposure-related QTc prolongation, and it is anticipated that QTc prolongation will be dose-limiting in this study. In order to reduce the risk of QTc prolongation, all efforts should be made to maintain the patient's serum potassium levels at >4 mmol/L during treatment with BGB324 and for 2 weeks following completion of therapy. Serum calcium and magnesium should be measured and corrected throughout treatment. Patients should not be discharged from the clinical site with a QTc of ≥480 ms. If this is the case, the patient's electrolytes should be measured and corrected as necessary.

If a patient experiences QTc prolongation despite normal serum potassium and magnesium levels, BGB324 dosing should be modified as outlined in Table 7.

Table 7: Dose Modification of BGB324 for QTc Prolongation

QTc value	Recommended dose modification
**QTc >450 ms at baseline**	Treatment not recommended
**QTc >500 ms (Grade 3) during treatment and change from pre-treatment value of <60 ms**	
1st occurrence	Temporarily interrupt treatment until QTc decreases <500 ms<br>Resume dosing at Reduced Dose Level (-1)<br>or resume dosing at Reduced Dose Level (-2) if the dose has already been lowered
2nd occurrence	Temporarily interrupt treatment until QTc decreases <500 ms<br>Resume dosing at Reduced Dose Level (-2)<br>or discontinue permanently if dose has already been lowered
3rd occurrence	Discontinue permanently
**QTc >500 ms and change for pretreatment value >60 ms**	
1st occurrence	Temporarily interrupt treatment until QTc decreases <500 ms<br>Resume dosing at Reduced Dose Level (-2)<br>or discontinue permanently if dose has already been lowered

Patients who resume BGB324 dosing after a temporary interruption for QTc prolongation should undergo more intensive ECG monitoring for the first four days, as if they were receiving BGB324 for the first time, as outlined in the relevant Schedule of Events (Tables 3-5).

11.2.2. Ara-C

11.3. Concomitant Medication and Procedures

All prescription, non-prescription, or over-the-counter medications including herbal remedies given to, or taken by, the patient at entry and during the study must be clearly documented in the Case Report Form (CRF).

The patients must be instructed that no additional medication will be allowed without the prior consent of the investigator. Any medication considered necessary for the patient's safety and well-being may be given at the discretion of the investigator. Hematopoetic growth factors may be administered for the treatment of AEs such as neutropenic infections but should not be used to maintain the dose intensity of BGB324 in Part A. Patients who have evidence of treatment benefit after ≥1 cycle of treatment in Part B may receive hematopoetic growth factors to maintain BGB324 dosing.

Concurrent treatment with any agent that may prolong the QT interval and may cause Torsade de Points is an exclusion criterion for the study. A comprehensive list of these prohibited medications is provided in Appendix 3.

11.4. Continuation of Treatment

Patients will continue to receive study treatment for as long as, in the opinion of the investigator, they continue to derive clinical benefit or until unacceptable toxicity, disease progression, death or withdrawal of consent.

11.5. Discontinuation of Treatment and Withdrawal of Patients

Patients may withdraw informed consent and discontinue from the study at any time, or for any reason, without prejudice to their future treatment. In addition a patient must be discontinued from the study if any of the following criteria are met:

- Patient non-compliance with the protocol, as agreed by the investigator and the sponsor
- Patient lost to follow-up
- Termination of the study by the sponsor
- Patient experiences an unacceptable toxicity that precludes them from continuing in the study, as agreed by the investigator and the sponsor
- Lack of recovery from a DLT to CTCAE Grade 0 or I within 14 days of occurrence, as outlined in Section 11.2
- Female patient becomes pregnant
- Patient experiences disease progression

AEs resulting in discontinuation will be followed as outlined in Section 8.3.4.

Patients who discontinue during the DLT assessment period (Part A), for any reason other than a DLT, will be replaced. All other patients who discontinue study treatment will not be replaced.

If a patient discontinues treatment prior to study completion, and for reasons other than progression or non-tolerability, every effort should be made to conduct the Final Study Visit assessments as outlined in Section 8.3.3. The reason for the patient's withdrawal from the study must be recorded in the CRF.

Patients with AML who do not achieve an objective response after 2 cycles of BGB324 monotherapy may start combination therapy with oral anti-metabolites (e.g. 6-mercaptopurine, hydroxyurea), low dose Ara-C or melphalan at approximately 50% of their institutional starting dose and the dose increased as tolerated.

12. Study Assessments

Study assessments will be conducted according to the relevant Schedule of Events (Tables 3-5) in Section 10.

12.1. Patient Selection Assays

In order to be eligible, patients with AML must have disease, which is sensitive to BGB324 in *ex vivo* culture as defined by IC_{50} of $\leq 2.5\ \mu M$

The eligibility of MDS patients will be based upon cell surface Axl expression on BM cells by FCM.

Patient selection assays will be performed centrally and detailed procedures for the collection, processing, storage and shipment of the samples will be provided in the Study Laboratory Manual.

12.1.1. BGB324 Sensitivity Assay

BM mononuclear cells will be analysed according to the methods described by *Ben-Batalla et al., 2013*.

12.1.2. Axl expression

BM mononuclear cells will be assessed for Axl expression by FCM.

12.2. Clinical Safety Assessments

Before being entered into the study, patients will be assessed to ensure that eligibility criteria are met. Patients not meeting the criteria must not be entered into the study.

The following tests and assessments will be performed at times specified in the relevant Schedule of Events (Tables 3-5) in Section 10 and at any other time required in the opinion of the investigator:

- Serum pregnancy test on women of child bearing potential (Screening only)

- Physical examination
- Vital sign measurements (systolic and diastolic BP, pulse, heart rate and temperature)
- Three serial 12-lead ECG within 5 minutes (should be performed after the patient has been supine for 5 minutes)
- AE and serious adverse event (SAE) recording
- Concomitant medication and procedures recording
- Echocardiogram

12.2.1. Resting 12-lead ECG

For timing of individual measurements please refer to relevant Schedule of Events (Tables 3-5) in Section 10.

Twelve-lead ECGs will be obtained after the patient has rested in a supine position for ≥5 minutes in each case. All 12-lead ECGs should be recorded while the patient is in the supine position. The investigator or designated physician will review the paper copies of each of the timed 12-lead ECGs on each of the study days when they are collected. ECGs will be performed in triplicate.

ECGs will be recorded at 25 mm/sec. A clinically significant ECG finding must be recorded as part of the medical history, if observed prior to start of dosing, and as an AE post -dose, where the finding represents a change from baseline.

12.2.2. Physical Examination

The physical examination will be symptom-directed and will exclude rectal and gynaecological (where applicable) examinations.

12.2.3. Vital signs

For timing of individual measurements please refer to relevant Schedule of Events (Tables 3-5) in Section 10.

BP and HR will be measured using a BP recording device with an appropriate cuff size. Measurements will be made after the patient has been resting supine for ≥5 minutes. Temperature and respiration rate will be measured as per clinical practice.

12.2.4. Subjective Symptomatology

Symptoms reported spontaneously by the patient will be recorded throughout the study period. Symptoms reported will be reviewed by the investigator and recorded as an AE where this is a change from baseline or a new symptom reported.

12.3. Laboratory Safety Assessments

Blood and urine samples will be taken at times specified in the relevant Schedule of Events (Tables 3-5) in Section 10, and at any other time required in the opinion of the investigator, and analysed at the designated local laboratory.

12.3.1. Haematology and Clinical Chemistry

Blood samples for the determination of clinical chemistry, hematology and coagulation parameters to be measured are outlined in Table 8.

Table 8: Haematology, Clinical Chemistry and Coagulation Parameters

Biochemistry	Haematology
Bilirubin	Haemoglobin
Direct and indirect bilirubin if total bilirubin is >ULN	Haematocrit<br>Erythrocytes
Aspartate aminotransferase (AST)	Mean cell haemoglobin (MCH)
Alanine aminotransferase (ALT)	Mean corpuscular haemoglobin concentration (MCHC)
Alkaline phosphatase (ALP)	Total white cell count
Creatine phosphokinase (CK)	Differential white cell count
Creatinine	Platelets
Creatinine Kinase (CK) and CK-Myocardial B (MB) if CK is elevated	**Coagulation**
Uric acid	Prothrombin time (PT)
Urea	Activated Partial Thromboplastin Time (APTT)
Albumin	
Total protein	
Potassium (K)	
Sodium (Na)	
Calcium (Ca)	
Chloride (Cl)	
Glucose	
Inorganic phosphate (IP)	

12.3.2. Serum Pregnancy Testing

No fertility or pregnancy studies have been conducted with BGB324 and therefore pregnant or lactating women are excluded from participating in this clinical study. Women of child-bearing potential must have a negative serum pregnancy test before BGB324 dosing can commence and must agree to practice one of the highly effective methods of contraception defined in Section 10.3. Serum pregnancy testing will be repeated every 6 weeks in this patient group throughout their participation in the study.

12.3.3. Urinalysis

Dipstick urinalysis for measurement of the parameters outlined in Table 9 will be conducted by the designated local laboratory:

Table 9: Urinalysis Parameters

pH<br>Glucose<br>Proteins<br>Ketones<br>Bilirubin<br>Urobilinogen	Nitrite<br>Blood<br>Leukocytes<br>Specific gravity<br>Urine microscopy (if indicated)

12.4. Pharmacokinetic Assessments

Venous blood samples for the determination of concentrations of BGB324 (Part A and Part B3_ and Ara-C (Part B3 ONLY) in plasma will be taken at times specified in the relevant Schedule of Events (Tables 3 and 5) in Section 10. Detailed procedures for the collection, processing, storage and shipment of the samples will be provided in the Study Laboratory Manual.

12.4.1. Determination of BGB324 Concentration in Plasma

Plasma samples for determination of BGB324 concentration will be analysed by BerGenBio's Bioanalytical Services vendor using the validated LC/MS/MS method.

12.4.2. Determination of Ara-C Concentration in Plasma

In Part B3 plasma samples for determination of Ara-C concentration will be analysed by the BerGenBio's Bioanalytical Services vendor using a validated method.

12.5. Clinical Efficacy Assessments

Clinical efficacy assessments will be performed once per cycle for Cycle $\geq$2, as outlined in the relevant Schedule of Events (Tables 3-5) in Section 10.

The assessment of the clinical efficacy of BGB324 in AML and MDS will be made, according to the recommendations of the IWG in AML [*Cheson et al., 2003*] and the modified IWG response criteria in MDS [*Cheson et al., 2006*] respectively, as follows:

- Assessment of the % of blasts in a BM aspirate sample
- Measurement of ANC
- Measurement of Platelet count $\geq$ 100 x 10^9/L
- Measurement of Hemoglobin concentration (MDS ONLY)
- Red cell transfusion requirement

BM aspirate and PBMC samples will be analysed at the designated local laboratory.

12.6. Pharmacodynamic Assessments

The effect of BGB324 on pharmacodynamic endpoints of axl inhibition is an exploratory objective for the study. Methods such as IHC, western blotting, ELISA, FCM, quantitative PCR, DNA-methylation analysis, transcriptomics, proteomics and genomics will be utilised.

- Effect of BGB324 on Axl and pAxl and downstream effectors of Axl signalling, to include but not limited to, Akt, pAkt, Erk, pErk, SLFN11, by appropriate methods (e.g. FCM, IHC, western blotting, proteomics, transcriptomics)
- Effect of BGB324 on gene expression by appropriate methods (e.g. transcriptomics, DNA-methylation analysis, proteomics, quantitative PCR, FCM, IHC, western blotting)
- Genomic analysis of normal tissue, and of cancer cells prior to and during treatment with BGB324, to investigate whether the spectrum of mutations present within the cancer cell population changes with BGB324 treatment

BM aspirates, PBMC and hair follicles samples will be collected at the time-points outlined in the relevant Schedule of Events (Tables 3-5) in Section 10. Detailed procedures for the collection, processing, storage and shipment of the samples will be provided in the Study Laboratory Manual.

12.6.1. Bone Marrow Aspirates

An aliquot of the BM aspirate samples collected for the assessment of clinical efficacy, as outlined in Section 12.5, will be processed for the PD investigation of Axl inhibition by BGB324. If the BM aspirate volume is insufficient, an additional PD blood sample will be taken.

- Effect of BGB324 on Axl and pAxl and downstream effectors of Axl signalling, to include but not limited to, Akt, pAkt, Erk, pErk, SLFN11 by appropriate methods (e.g. western blotting, proteomics, transcriptomics)
- Genomic analysis of normal tissue, and of cancer cells prior to and during treatment with BGB324, to investigate whether the spectrum of mutations present within the cancer cell population changes with BGB324 treatment

12.6.2. Blood Samples

Blood samples will be collected at the time-points outlined in the relevant Schedule of Events (Tables 3-5) in Section 10.

- Effect of BGB324 on Axl and pAxl and downstream effectors of Axl signalling, to include but not limited to, Akt, pAkt, Erk, pErk, SLFN11 by appropriate methods (e.g. FCM, IHC, western blotting, proteomics, transcriptomics)

As outlined in Section 12.6.1, if the BM aspirate volume proves insufficient for PD analysis, an additional PD blood sample will be taken.

12.6.3. Hair Follicle Samples

Hair follicle samples will be collected at the time-points outlined in the relevant Schedule of Events (Tables 3-5) in Section 10. The hair follicle samples will be analysed for:

- Effect of BGB324 on Axl and pAxl and downstream effectors of Axl signalling, to include but not limited to, Akt, pAkt, Erk, pErk, SLFN11 by appropriate methods (e.g. FCM, IHC, western blotting, proteomics, transcriptomics)

12.6.4. Normal Tissue Samples

A skin biopsy or hair follicles, according to local practice, will be taken pre-dose on Cycle 1 for PD biomarker genomics analysis:

- Genomic analysis of normal tissue, and of cancer cells prior to and during treatment with BGB324, to investigate whether the spectrum of mutations present within the cancer cell population changes with BGB324 treatment

12.7. Volume of Blood Sampling

The volume of blood that will be drawn from each patient per cycle is detailed in the tables below:

Table 10: Volume of blood and BM aspirate to be drawn per Patient during Screening and at Cycle 1

**Assessment**		**Sample volume (mL)**	**Part A**		**Parts B1 & B2**		**Part B3**	
			**n**	**Total volume (mL)**	**n**	**Total volume (mL)**	**n**	**Total volume (mL)**
**Blood**								
Pharmacokinetics		3	21	63	NA	NA	28	84
Pharmacodynamics		3.5	21	73.5	8	28	21	73.5
Coagulation screen		5	4	20	4	20	4	20
Safety	Clinical chemistry	5	13	65	13	65	13	65
	Hematology	2.5	13	32.5	13	32.5	13	32.5
**Total blood for screen and Cycle 1**			**254**		**130.5**		**275**	
**BM aspirate**								
Pharmacodynamics		19	2	38	2	38	2	38
Clinical efficacy assessment		1	2	2	2	2	2	2
**Total BM for screen and Cycle 1**			**40**		**40**		**40**	

Table 11: Volume of Blood and BM aspirate to be drawn per Patient during Cycle 2

Assessment		Sample volume (mL)	Part A		Parts B1 & B2		Part B3	
			n	Total volume (mL)	n	Total volume (mL)	n	Total volume (mL)
**Blood**								
Pharmacokinetics		3	3	9	NA	NA	3	9
Pharmacodynamics		3.5	3	10.5	3	10.5	3	10.5
Coagulation screen		5	1	5	1	5	1	5
Safety	Clinical chemistry	5	3	15	3	15	3	15
	Hematology	2.5	3	7.5	3	7.5	3	7.5
**Total blood for screen and Cycle 1**			**47**		**38**		**47**	
**BM aspirate**								
Pharmacodynamics		**19**	**1**	**19**	**1**	**19**	**1**	**19**
Clinical efficacy assessment		1	1	1	1	1	1	1
**Total BM for screen and Cycle 1**			**20**		**20**		**20**	

Table 12: Volume of Blood and BM aspirate to be drawn per Patient per Cycle ≥3 & at Final Study Visit

Assessment		Sample volume (mL)	Part A		Parts B1 & B2		Part B3	
			n	Total volume (mL)	n	Total volume (mL)	n	Total volume (mL)
**Blood**								
Pharmacokinetics		3	1	3	NA	NA	1	3
Pharmacodynamics		3.5	1	3.5	1	3.5	1	3.5
Coagulation screen		5	1	5	1	5	1	5
Safety	Clinical chemistry	5	1	5	1	5	1	5
	Hematology	2.5	1	2.5	1	2.5	1	2.5
**Total blood per Cycle & Final Visit**			**12.5**		**12.5**		**12.5**	
**BM aspirate**								
Pharmacodynamics		19	1	19	1	19	1	19
Clinical efficacy assessment		1	1	1	1	1	1	1
**Total BM per Cycle & at Final Visit**			**20**		**20**		**20**	

13. Study Treatment Management

Every patient enrolled in the study will receive BGB324. Patients enrolled in Part B3 will receive BGB324 in combination with low dose Ara-C.

13.1. Study Medication

The investigational medicinal product (IMP) BGB324 must be stored in a secure location. Accountability for study treatment is the responsibility of the investigator. The investigator/designee must ensure that the IMP will only be dispensed to patients in accordance with the protocol and that any unused IMP will be disposed of or, if unopened, returned in accordance with the written instructions of the sponsor.

Study staff should refer to the sponsor's Directions for Handling and Administration (DHA) document for specific instructions regarding the handling, storage, dispensing and destruction of the IMP.

BGB324 has been manufactured according to appropriate Good Manufacturing Practice (GMP) standards. BGB324 will be labelled in compliance with GMP Annex 13 requirements and local regulatory guidelines.

13.1.1. BGB324

BGB324 will be supplied in size zero Swedish orange hydroxypropyl methylcellulose (HPMC) capsules at a dose strength of 100 mg for oral dosing. Please refer to Section 4 of the current version of the IB for additional information on the physical, chemical and pharmaceutical properties of BGB324.

13.1.1.1. BGB324 Storage

BGB324 will be shipped to the site and must be stored at the site in a secure location under ambient temperature conditions.

Detailed instructions for the storage of dispensed IMP at home will be provided to every patient enrolled in the clinical study.

13.1.1.2. BGB324 Accountability

The investigator/designee must maintain complete and accurate IMP accountability records showing the date of receipt and quantity of all supplies of IMP. These records must include accurate patient-specific dispensing information including quantity of capsules/bottles dispensed, date dispensed and quantity and date returned (or disposed of).

At the end of the study reconciliation must be made between the amount of IMP supplied, dispensed and subsequently returned to the sponsor or destroyed at site and any discrepancies accounted for.

Destruction of the IMP at site will only be performed by authorised personnel following written receipt of approval from the sponsor. The procedure should be fully documented as outlined in the DHA.

13.1.2. Ara-C

All patients in Part B3 will receive subcutaneous Ara-C per standard practice.

Name: Cytarabine (*Generic product*)

Brand name: Ara-C

Active Substance: Cytosine Arabinoside

Pharmaceutical form: Solution for injection

Route of administration: Subcutaneous

Investigational Medicinal Product Status: Non-IMP

ATC code: L01BC01

Ara-C is a generic product and can be produced by multiple manufactures. Ara-C will be packaged and labelled in the standard manner according to the current marketing authorisation by the manufacturer.

Ara-C will be purchased by the participating hospital via their normal purchasing procedure and will not be reimbursed by BerGenBio AS.

13.1.2.1. Ara-C Storage

Ara-C will be stored in accordance with the specific manufacturer's SmPC. A copy of the SmPC for the product used should be kept in the Pharmacy file.

13.1.2.2. Ara-C Accountability

The investigator/designee must maintain complete and accurate Ara-C dispensing logs including the actual dose administered and the date.

14. Safety Definitions, Monitoring and Reporting

Safety reporting in this clinical study will follow the "Detailed Guidance on the collection, verification and presentation of adverse reaction reports arising from clinical trials on medicinal products for human use (CT-3)'' presented in 2011/C 172/01.

14.1. Definitions

The following definitions are according to the Directive 2001/20/EC.

14.1.1. Adverse Events

An AE is any untoward medical occurrence in a patient or clinical study subject administered a pharmaceutical product and which does not necessarily have a causal relationship with the product. An AE can therefore be any unfavourable or unintended sign, symptom or disease

temporally associated with the use of the IMP whether or not considered related to the IMP. This includes any occurrence that is new, an exacerbation of an existing disease (a worsening of the character, frequency or severity of a known condition) or abnormal results of diagnostic procedures, including laboratory test abnormalities, with the exception of events that are clearly consistent with the pattern of leukemia progression.

14.1.2. Adverse Reaction

An adverse reaction (ADR) **is** defined as all untoward and unintended responses to an IMP related to any dose administered. The definition implies a reasonable possibility of a causal relationship between the event and the IMP i.e. there are facts (evidence) or arguments to suggest a causal relationship.

14.1.3. Serious Adverse Events

A **serious adverse event** (SAE) as defined by International Council for Harmonisation (ICH) (Section 1.50) is any untoward medical occurrence that meets any of the following conditions:

- Results in death
- Is life-threatening (i.e. the patient was at risk of death at the time of the event; it does not refer to an event which hypothetically might have caused death if it were more severe)
- Requires in-patient hospitalisation or prolongation of existing hospitalisation
- Results in persistent or significant disability/incapacity
- Results in a congenital anomaly/birth defect

Important medical events that may not be immediately life-threatening or result in death or hospitalisation but may jeopardise the patient or may require intervention to prevent one of the other outcomes listed in the definition above, should also be considered serious.

14.2. Monitoring and Reporting Events

14.2.1. Adverse Events

Any AE experienced by a patient between signing the informed consent form (ICF) and 28 days after the last administration of BGB324 is to be recorded in the CRF regardless of the severity and causality of the event.

14.2.2. Serious Adverse Events

Any SAE experienced by a patient between the time of the first administration of BGB324 and 28 days after the last administration is to be recorded on an SAE Report Form within 24 hours of receipt, regardless of the severity and causality of the event. The SAE Report Form

should be faxed or e-mailed to the Ockham Drug Safety group. A telephone report may only be made in exceptional circumstances and must be followed by completion of the SAE Report Form within 1 working day. Where applicable, information from relevant hospital case records and post-mortem reports should be obtained.

All SAEs that have not resolved by the end of the study, or that have not resolved upon the patient's discontinuation from the study, must be followed until any of the following occur:

- The event resolves
- The event stabilises
- The event returns to baseline status
- The event can be attributed to agents other than the IMP or to factors unrelated to study conduct
- It becomes unlikely that any additional information can be obtained

Any event requiring hospitalisation (or prolongation of hospitalisation) that occurs during the course of a patient's participation in a clinical study must be reported as an SAE. Exclusions to this are hospitalisations:

- For pre-scheduled or routine elective procedures (as long as the qualifying condition did not worsen or progress, in the opinion of the investigator, since signing the ICF)
- For social reasons, in the absence of an AE e.g. for respite care
- Due to expected deterioration caused by disease progression

14.2.3. All Events

All events must be assessed to determine the following:

- If the event meets the criteria for an SAE as defined in Section 14.2.2
- The severity of the event as defined in Section 14.3.1
- The relationship to BGB324 as defined in Section 14.3.2

14.3. Safety Classifications

14.3.1. Assessment of Severity

The investigator/designee will rate the severity of each AE according to CTCAE. For each sign or symptom, the maximum or highest grade observed since the last visit should be reported. If the symptom or event cannot be graded according to CTCAE, the following grade (severity) rating guideline will be used:

**Grade 1**	Mild	The AE is transient and easily tolerated by the subject

**Grade 2**	Moderate	The AE causes the subject discomfort and interrupts the subject's usual activities
**Grade 3 or 4**	Severe	The AE causes considerable interference with the subject's usual activities and may be incapacitating or life-threatening

14.3.2. Assessment of Causality

The investigator will assess the causality of the AE with respect to the IMP according to the following categories derived from the World Health Organisation (WHO) Collaborating Centre for International Drug Monitoring:

Not related	An AE is due to an underlying or concurrent illness or effect of another drug and is not related to the IMP e.g., has no temporal relationship to the IMP or has a much more likely alternative aetiology
Suspected	An AE has a strong temporal relationship to the IMP and an alternative aetiology is equally or less likely, compared to the potential relationship to the IMP OR<br>An AE has a strong temporal relationship to the IMP or recurs on re-challenge and another aetiology is unlikely or significantly less likely

If in the opinion of the investigator an SAE is considered not related an alternate aetiology must be provided. The causality assessment given by the investigator should not be downgraded by the sponsor. If the sponsor disagrees with the investigator's causality assessment, both the opinion of the investigator and the sponsor should be provided with the report.

14.3.3. Assessment of Expectedness

An **unexpected adverse event** is an event, the nature or severity of which is not consistent with the reference safety information (RSI) presented in the IB for the IMP. The responsibility for determining expectedness of an ADR falls to the sponsor.

14.3.4. Immediate Reporting of Serious Adverse Events

Expedited safety reporting within this clinical study complies with the ICH Guideline for Clinical Safety Data Management: Definitions and Standards for Expedited Reporting (E2A), investigational new drug (IND) safety reporting and safety reporting (under 21 CFR 312.32) and with applicable local regulatory requirements.

The sponsor assumes responsibility for appropriate reporting of AEs to the Regulatory Authorities and main Research Ethics Committee (REC)/Institutional Review Board (IRB). Although this responsibility can be delegated to the sponsor's Contract Research organisation (CRO), the sponsor retains ultimate responsibility for safety reporting.

All SAEs that are unexpected and associated with the use of the IMP are classified as suspected unexpected serious adverse reactions (SUSARs) and must be reported to the main REC/ IRB and Regulatory Authorities within 7 calendar days if fatal or life threatening, or within 15 calendar days if non-life threatening and non-fatal. The sponsor/designee must also report SUSARs to all investigational sites.

Any safety information from other observations that could change the risk-benefit evaluation of the product should be promptly communicated to the Regulatory Authorities and main REC/IRB. Any other SUSARs associated with the IMP should be reported as soon as the sponsor becomes aware of them including SUSARs which occur in another clinical study conducted by the same sponsor or which are identified by spontaneous reports, a publication, or which are transmitted to the sponsor by another Regulatory Authority.

Other safety issues that also qualify for expedited reporting where they might materially alter the current benefit-risk assessment of the IMP (sufficient to consider changes in the administration or in the overall conduct of the trial), for instance include:

- An increase in the rate of occurrence of an expected serious adverse reaction, which is judged to be clinically important
- Post-study SUSARs that occur after the patient has completed a clinical trial and are reported by the investigator to the sponsor
- New events relating to the conduct of the trial or the development of the IMP likely to affect the safety of the patients, such as:
    - An SAE which could be associated with the trial procedures and which could modify the conduct of the trial
    - A major safety finding (such as carcinogenicity, which differs to the underlying disease)

Expedited reporting is not usually required for reactions which are serious but expected, and it is inappropriate to report events that are considered unrelated to the IMP.

14.3.5. Annual Safety Updates

Once a year the sponsor/designee will submit an annual Development Safety Update Report (DSUR) to the Regulatory Authorities and main REC/IRB that lists all suspected serious adverse reactions occurring throughout the time period.

14.3.6. Deaths

The cause of death of a patient in a clinical study, unless it is associated with disease progression, is considered a SAE whether the event is expected or associated with the IMP.

14.4. Procedures for Handling Special Situations

14.4.1. Pregnancy

As detailed in the inclusion/exclusion criteria, patients of childbearing potential who are not using adequate contraception will not be invited to participate in this study. Female patients of childbearing potential must have a negative serum pregnancy test within 3 days prior to the first dose of BGB324. In the event of any pregnancies occurring in female patients, or in the partners of male patients during the study, the pregnancy must be reported to the Ockham Drug Safety group by the investigational staff within one working day of their knowledge of the event using a Pregnancy Notification Form.

The reporting period for pregnancies will start with the first study-related procedure and end 28 days after the final administration of BGB324.

Any female patients who become pregnant whilst taking part in the study will be withdrawn, whilst patients whose partners become pregnant can remain in the study. A patient who completes or withdraws from the trial before the full term of their/their partner's pregnancy will be asked to consent to their doctor providing the sponsor with follow-up information concerning the pregnancy and its outcome.

14.4.2. Medical Emergency

In a medical emergency requiring immediate attention, study staff will apply appropriate medical intervention according to standard hospital practice. The investigator/designee will contact BerGenBio's Medical Monitor.

14.4.3. Unblinding for a Medical Emergency

Not applicable.

14.5. Investigator's Safety Responsibilities

The investigator's responsibilities include the following:

- Monitor and record all AEs, including SAEs, regardless of the severity or relationship to BGB324
- Determine the seriousness, relationship and severity of each event
- Determine the onset and resolution of each event
- Complete an SAE form for each SAE as outlined in Section 14.2.2
- Pursue SAE follow-up information actively and persistently

- Ensure all AE and SAE reports are supported by documentation in the patient medical records
- Report SAEs to their local REC/IRB, as required by local law

14.6. Sponsor's Safety Responsibilities

The sponsor's responsibilities include the following:

- The ongoing safety evaluation of the IMP
- Reporting of SUSARs to the Regulatory Authorities, main REC/IRB and investigators according to required timelines as outlined in Section 14.3.4
- Submission of the annual DSUR to the Regulatory Authorities and main REC/IRB

15. Statistical Methods And Determination Of Sample Size

Detailed statistical analysis information will be provided separately in the Statistical Analysis Plan (SAP). Any deviations to the planned analyses specified within the SAP, will be justified in writing and presented within the final clinical study report (CSR).

In the Tables, Listings and Figures in the CSR, patients enrolled in Part A will be reported separately. Where appropriate, the data for patients who received the MTD during Part A will be combined with that for patients who received the MTD in Part B for the purpose of data summarisation. A clear distinction will be made in the relevant Tables to differentiate between the cohort who received the MTD during the dose escalation phase of Part A, the MTD cohort from the cohort expansion phase in Part A, and the combined MTD cohort.

If necessary, after the clinical phase of the study has been completed, a decision will be made between the sponsor and the study statistician regarding management of missing values, data for withdrawn patients and data for protocol violators.

The database lock will take place when all data are reconciled. A single CSR will be generated for this study.

15.1. Description of Objectives

Please refer to Section 7.1.

15.2. Description of Endpoints

Please refer to Section 7.2.

15.3. Demography and Baseline Disease Characteristics

The Enrolled Population will include all patients who have signed the ICF and enrolled into the study. The enrolment date will be defined as the date of the first protocol-defined screening procedure. All patients in the Enrolled Population will be accounted for in patient disposition. Patient disposition will be summarised by dose group. Medical history and prior medications will be tabulated.

15.4. Safety

15.4.1. Analysis Population

The safety analysis set (SAS) will be defined as all patients who receive at least one dose of BGB324. The SAS will be analysed by dose group.

15.4.2. Methods of Analysis

No formal statistical analysis will be performed on safety data. AEs will be coded using the Medical Dictionary for Regulatory Activities (MedDRA) Version 15.0 or above. The number and percentage of patients reporting treatment-emergent AEs will be tabulated by preferred term and system organ class, and summarized by both CTCAE grade and relationship to study drug. All AEs commencing prior to dosing with study medication will be excluded from the tabulation but will be fully listed. AEs that have missing onset dates will be considered to be treatment-emergent, unless the stop date is known to be prior to the first administration of BGB324.

Separate listings will be produced for DLTs, SAEs, discontinuations due to related AEs, and events of >Grade 3 severity.

Summary statistics by dose cohort and time point will be produced for vital signs, ECG parameters and laboratory safety data, together with changes from baseline. The incidence of laboratory test results outside the normal range will be summarized by dose cohort and time point. Laboratory test results outside the normal range will be individually listed for clinical review. Abnormal ECG results will be summarized by dose cohort.

Changes in PR, QT and QTcF duration will be analysed according to administered dose and ultimately modelled with corresponding plasma concentrations of BGB324 when available.

15.5. Efficacy

15.5.1. Analysis Population

The Efficacy Analysis Population will include all subjects who receive $\geq$1 dose of BGB324.

15.5.2. Methods of Analysis

Although all efficacy analysis for this study is to be regarded as essentially exploratory, there will be formal hypothesis tests for the objective response rate (ORR). These will consist of one-sided, within-group tests of proportion of responders for each criterion, against the null hypothesis of a response rate $\leq$5% (this being the observed response rate under current treatment). Values of P <0.2 will be taken as sufficient evidence of a trend to justify further study. Patients who on review do not meet the protocol inclusion/exclusion criteria may be excluded from the efficacy analysis.

Data relating to efficacy response will be listed and summarized by dose cohort and time point, as appropriate.

15.6. Pharmacokinetics

15.6.1. The Analysis Population

The Pharmacokinetics Analysis Population will include all patients who receive ≥1 dose of BGB324 and have evaluable PK data available.

15.6.2. Methods of Analysis

The PK parameters listed in Table 13 (but not limited to) will be derived from the relevant concentration data of BGB324 (Part A and Part B3) and Ara-C (Part B3 only) by non-compartmental analysis using WinNonlin Phoenix Version 6.3 (or higher version). Full details of the PK methodology will be written in a PK Analysis Plan.

Table 13: Plasma PK parameters

C_{max}	The observed maximum plasma concentration after single dose administration
t_{max}	The time to reach C_{max}
$AUC_{0\text{-}tau}$	The area under the curve within a dosing interval, calculated by the linear up-log down trapezoidal method.
λ_z	The apparent terminal rate constant estimated from individual linear regression of the terminal part of the log concentration vs. time curve
$t_{½}$	The apparent terminal half-life, calculated by $0.693/\lambda_z$

Where possible, individual, mean and median BGB324 (and Ara-C) plasma concentration-time data will be presented in tabular and/or graphical form.

15.7. Pharmacodynamics

15.7.1. The Analysis Population

The Pharmacodynamic Analysis Population will include all patients who receive ≥1 dose of BGB324 and have ≥1 sample collected for analysis.

15.7.2. Methods of Analysis

All pharmacodynamic biomarkers will be listed and summarised by dose cohort and time-point as appropriate.

15.8. Safety Monitoring Committee

No formal statistical interim analyses are planned.

Data will be monitored by the SMC, in an on-going manner, to assess patient safety as outlined in Section 8.2.1.1. During Part A, the SMC will be responsible for:

- Reviewing the safety data from Cycle 1 of each dose cohort to determine whether dose escalation can proceed
- Determining the MTD prior to commencing Part B
- Defining the starting dose of BGB324 to be administered with Ara-C in Part B3

During the execution of Part B the SMC will convene after 10 patients have completed Cycle 1 of treatment

15.9. Sample Size Considerations

Part A of the study follows a conventional algorithm (3+3 patients per dose level) to identify the MTD, escalating on 0/3 or 1/6 patients experiencing a DLT. Under this design, there is a 71% chance of escalation if the true but unknown rate of DLT is 20%, and <50% chance of escalation if the true but unknown rate of DLT is >30%. The operating characteristics of this 3+3 design are outlined in Table 14.

Table 14: Operating Characteristics of the 3+3 Study Design

True but Unknown Rate of DLT (%)	Probability of Escalation (%)
20	71
30	49
40	31
50	17
60	8

The total sample size of Part A depends upon whether DLT(s) is experienced at a given dose level (i.e. whether 3 or 6 patients have been treated) and how many dose levels are tested in order to reach the MTD. A total of 10 patients will be treated at the MTD.

The sample size of 16 patients for Parts B1, B2 and B3 in this study has been calculated on the basis of the following assumptions for a one-sided, within-group test of proportions, comparing an anticipated ORR of $\geq$20% against the null hypothesis rate of 5%, with power 80% for a test of size 0.2. This calculation is based on statistical significance at the 0.2 level, since this will be taken as evidence of a trend worthy of further study. Although this is offered as a formal sample size calculation, it is acknowledged that for this early-stage study the analyses will be exploratory in nature, and that no account has been taken of multiplicity inherent in the assessment of several, presumably non-independent criteria, for ORR.

15.10. Clinical Study Report

The results of the study will be presented in an integrated clinical study report according to ICH guidelines.

16. Ethical Requirements

16.1. Good Clinical Practice Statement

The clinical study will be performed in accordance with the protocol, the Declaration of Helsinki, ICH Guidelines, Good Clinical Practice (GCP), the European Union (EU) Clinical Trial Directive (CTD) 2001/20/EC Guideline for GCP CPMP/ICH/135/95, and all applicable local regulatory requirements.

Clinical Trial Authorization (CTA) will be obtained from the relevant Regulatory Authority prior to initiation of the study within a specific country.

16.2. Ethics Committee

The final study protocol and ICF must be approved, in writing, by the relevant REC/IRB before patient enrolment commences.

The following information should be reported to the REC/IRB during the study:

- All amendments to the research (substantial and non-substantial amendments)
- Annual progress reports
- Individual SUSARs
- DSUR on an annual basis
- Urgent Safety Measures

The investigator/designee must notify the REC/IRB within 90 days of completion of the study. However if the study is terminated prematurely, written explanation of the reason(s) for the early termination must be provided within 15 days.

16.3. Patient Information and Consent

Prior to performing any study-related activities, including screening tests and procedures, written informed consent must be obtained from the patient, in accordance with local regulations.

The ICF must be in accordance with the principles that originated in the Declaration of Helsinki, current ICH and GCP guidelines, applicable regulatory requirements and sponsor policy and must be approved by the reviewing REC/IRB.

16.4. Subject Data Protection

Personally identifiable information (PII) i.e. identifiable information from or about an individual will not be collected by the sponsor. The collection and processing of personal data from patients enrolled in this study will be limited to those data that are necessary to investigate the efficacy, safety, tolerability, quality and utility of the IMP used in this study. These data must be collected and processed with adequate precautions to ensure confidentiality and compliance with applicable data privacy protection laws and regulations.

The ICF must explain that identifying information will be kept on file and that portions of the patient's medical records pertinent to the study will be reviewed by sponsor personnel (or their designees) and possibly by Regulatory Agencies and the REC/IRB for data verification purposes.

17. Administrative Procedures

17.1. Quality Assurance

In compliance with GCP and regulatory requirements, the sponsor/designee, external Regulatory Agency and/or REC/IRB may conduct quality assurance (QA) audits at any time during or following completion of the study. The investigator will be expected to cooperate with any audit and to provide assistance and documentation (including source data) as requested.

17.2. Case Report Form and Source Document Verification

Electronic CRFs will be provided for recording clinical data. Designated site personnel will record data in the eCRF for observations, tests and assessments as specified in the protocol. The investigator must permit direct access to designated source documentation (e.g. medical records) for the purpose of verifying that the data recorded in the eCRF are consistent with the original source data.

17.3. Study Monitoring

Before a patient can be screened for enrolment into the study, the sponsor/designee will conduct a study initiation visit to:

- Determine that the site facilities are adequate
- Discuss responsibilities with regard to the protocol with the investigator and study team
- Conduct training on completion of the eCRF and other study related documentation
- Review study procedures

During the conduct of the study, the sponsor/designee will provide support to the investigator and study team via telephone/e-mail communication. The sponsor/designee will conduct regular site visits, according to applicable ICH and GCP guidelines, to:

- Confirm the facilities remain acceptable
- Confirm the site study team are adhering to the protocol and regulatory requirements
- Confirm that the IMP is being properly maintained and accountability records are accurate and current
- Confirm data are being accurately recorded in the eCRF by performing source document verification

17.4. Notification of Serious Breaches

In accordance with Regulation 29A of the Medicines for Human Use (Clinical Trials) Regulations 2004 and its amendments, and where this is a requirement according to local

law, the sponsor of the trial is responsible for notifying the Regulatory Authorities in writing, within 7 days, of any serious breach of:

- The conditions and principles of GCP in connection with the study
- The protocol relating to the study

Whilst under investigation, the site is requested to cooperate with the sponsor/designee in providing sufficient information to report the breach to the Regulatory Authorities where required, and in undertaking any corrective and/or preventive action.

17.5. Retention of Study Data

A copy of all records and essential documents must be retained in the study files as follows (whichever is the longest period): for a minimum of two years following the last approval of the drug in an ICH region and until there are no pending or contemplated marketing applications in an ICH region: a minimum of two years have elapsed since the formal discontinuation of the clinical development of the drug: or for the minimum length of time after the clinical study has ended (in accordance with local regulations). No documents should be destroyed without prior written agreement between the sponsor and the investigator.

Should the investigator wish to assign the documents to another party or move them to another location, the sponsor must be notified. The sponsor will notify the investigator in writing when the study-related documents are no longer needed.

17.6. Use of Information and Publication Policy

All information regarding BGB324 and the sponsor's operations (e.g. patent applications, formulas, manufacturing processes, basic scientific data, or formulation information) supplied by the sponsor to the investigator and not previously published is considered confidential. This confidential information remains the sole property of the sponsor and shall not be disclosed to others without the written consent of the sponsor. The investigator agrees to use this information only to perform this study and will not use it for other purposes including publications and presentations without the sponsor's written consent. The full terms of confidentiality, intellectual property and publication policy are detailed in the current Clinical Trial Agreement between the sponsor and the site.

17.7. Contract Research Organisations

Data management, safety reporting, statistics and programming, auditing and QA services will be outsourced to the sponsor's CRO Ockham Oncology.

PK analyses will be outsourced to the sponsor's Bioanalytical services vendor.

17.8. Changes to the Final Study Protocol

All protocol amendments must be submitted to the REC/IRB and, if applicable, to the Regulatory Authorities.

Protocol amendments that affect patient safety, the scope of the investigation, or the scientific quality of the study should not be implemented without prior Regulatory Authority/REC/IRB approval, except where necessary to eliminate immediate hazards to the patients.

17.9. Investigator Responsibilities

By signing the protocol the investigator agrees to:

- Conduct the study in accordance with the protocol and make changes only after notifying the Sponsor, except to protect the safety, rights or welfare of patients
- Personally conduct or supervise the study
- Inform any patients enrolled in the study that the drug is being used for investigational purposes
- Ensure that the requirements relating to obtaining informed consent and REC/IRB review and approval meet Federal guidelines, as stated in 21 CFR, parts 50 and 56
- Report to the Sponsor any AEs that occur during the course of the study, in accordance with 21 CFR 312.32, as well as ICH guidelines
- Have read and understood the IB for BGB324, including potential risks and side effects of the drug
- Ensure that all associates, colleagues and employees assisting in the conduct of the study are informed about their obligations in meeting the above commitments
- Maintain adequate and accurate records, in accordance with 21 CFR 312.62 and to make those records available for inspection by the sponsor, their designated representative, relevant Regulatory Authorities/REC/IRB or any agency authorized by law
- Ensure that a REC/IRB that complies with the requirements of 21 CFR 56 will be responsible for initial and continuing review and approval of the clinical study
- Report promptly to the REC/IRB and the sponsor all changes in the research activity and all unanticipated problems involving risks to subjects or others (to include amendments and IND safety reports)
- Not make any changes in the research study without approval, except when necessary to eliminate hazards to the subjects/subjects
- Comply with all other requirements regarding the obligations of the clinical investigators and all other pertinent requirements listed in 21 CFR 312

18. References

Ben-Batalla I, Schultze A, Wroblewski M, et al., Axl a Prognostic and Therapeutic Target in Acute Myeloid Leukemia mediates Paracrine Crosstalk of Leukemia Cells with Bone Marrow Stroma. *Blood* (2013) 122: 2443 - 2452

Cheson BD, Bennett JM, Kopecky KJ, *et al.*, Revised Recommendations of the International Working Group for Diagnosis, Standardisation of Response Criteria, treatment Outcomes and Reporting Standards for Therapeutic Trials in Acute Myeloid Leukemia. *J ClinOncol* (2003) 21 (24): 4642 - 4649

Cheson BD, Greenberg PL, Bennett JM *et al.*, Clinical Application and Proposal for Modification of the International Working Group (IWG) Response Criteria in Myelodysplasia. *Blood* (2006) 108 (2): 419 - 425

Greenberg PL, Cox C, LeBeau MM, *et al.* International Scoring System for Evaluating Prognosis in Myelodysplastic Syndromes. *Blood* (1997) 89 (6): 2079 – 2088

Oken MM, Creech RH, Tormey DC, *et al.* Toxicity and Response Criteria of the Eastern Cooperative Oncology Group. *Am J Clin Oncol* (1982) 5: 649 - 655

Whitman SP, Kohlschmidt J, Maharry K, *et al.,* Gas6 Expression Identifies High-Risk AML Patients: Potential Implications for Therapy. *Leukemia* (2013) Dec 11. doi: 10.1038/leu.2013.371. [Epub ahead of print]

The Criteria Committee of the New York Heart Association. Nomenclature and Criteria for Diagnosis of Diseases of the Heart and Great Vessels. (1994). (9th ed.). Boston: Little, Brown & Co. 253 – 256

19. Appendices

Appendix 1: Eastern Cooperative Oncology Group (ECOG) Performance Status

Grade	ECOG
0	Fully active, able to carry on all pre-disease performance without restriction
1	Restricted in physically strenuous activity but ambulatory and able to carry out work of a light or sedentary nature, e.g., light house work, office work
2	Ambulatory and capable of all selfcare but unable to carry out any work activities. Up and about more than 50% of waking hours
3	Capable of only limited selfcare, confined to bed or chair more than 50% of waking hours
4	Completely disabled. Cannot carry on any selfcare. Totally confined to bed or chair
5	Dead

From *Oken et al., 1982* with credit to the ECOG Chair Robert Comis M.D

Appendix 2: The New York Heart Association (NYHA) Functional Classification in a Patient with Heart Disease

Overview: The New York Heart Association (NYHA) developed a functional classification for patients with heart disease.

Patients: Heart disease must be present.

Parameters:

1) Limitations on physical activity
2) Symptoms (undue fatigue palpitations dyspnea and/or anginal pain) with ordinary physical activity
3) Status at rest

Limitations on Physical Activity	Symptoms with Ordinary Physical Activity	Status at Rest	Class
none	none	comfortable	I
slight	symptomatic with ordinary activities	comfortable	II
marked	symptomatic at less than ordinary levels of activity	comfortable	III
unable to perform any activity	discomfort with any activity	symptomatic at rest	IV

From The Criteria Committee of the New York Heart Association. Nomenclature and Criteria for Diagnosis of Diseases of the Heart and Great Vessels. (1994). (9th ed.). Boston: Little, Brown & Co. 253 – 256

Appendix 3: Summary of Drugs that are Generally Accepted to Have a Risk of Causing QTc Prolongation Potentially Causing Torsades de Pointes

Generic Name	Brand Name
Amiodarone	Coradone/Pacerone
Aresenic trioxide	Trisenox
Astemizole	Hismanal
Bepridil	Vascor
Chlorquine	Arelan
Chlorpromazine	Thorazine
Cisapride	Propulsid
Clarithromycin	Biaxin
Disopyramide	Norpace
Dofetilide	Tikosyn
Domperidone	Motilium
Droperidol	Inapsine
Erythromycin	Erythrocin/E.E.S.
Halofantrine	Halfan
Haloperidol	Haldol
Ibutilide	Covert
Levomethadyl	Orlaam
Mesoridazine	Serentil
Methadone	Methadose/Dolophine
Moxifloxacin	Avelox
Petamidine	NebuPent/Pentam
Pimozide	Orap
Probucol	Lorelco
Procainamide	Pronestyl/Procan
Quinidine	Cardioquin/Quinaglute
Sotalol	Betapace
Sparfloxacin	Zagam
Terfenadine	Seldane
Thioridazine	Mellaril
Vandetanib	Zactima

Adapted from "The University of Arizona Center for Education and Research on Therapeutics"

See the following website for an updated list of drugs that prolong QT and/or cause Torsades de Pointes

http://crediblemeds.org/everyone/composite-list-all-qtdrugs/?rf=All
