# Supplementary material for: Bemcentinib as monotherapy and in combination with low-dose cytarabine in acute myeloid leukemia patients unfit for intensive chemotherapy: a phase 1b/2a trial
Source: Nat Commun. 2025 Mar 23;16:2846. doi: 10.1038/s41467-025-58179-6 (PMC11930985; doi:10.1038/s41467-025-58179-6)
Supplement: Supplementary file 2 — Reporting Summary [file 41467_2025_58179_MOESM2_ESM.pdf]

nature portfolio

Corresponding author(s): Sonja Loges

Last updated by author(s): Feb 24, 2025

Reporting Summary

Nature Portfolio wishes to improve the reproducibility of the work that we publish. This form provides structure for consistency and transparency in reporting. For further information on Nature Portfolio policies, see our Editorial Policies and the Editorial Policy Checklist.

Statistics

For all statistical analyses, confirm that the following items are present in the figure legend, table legend, main text, or Methods section.

n/a	Confirmed	
☐	☒	The exact sample size (*n*) for each experimental group/condition, given as a discrete number and unit of measurement
☒	☐	A statement on whether measurements were taken from distinct samples or whether the same sample was measured repeatedly
☐	☒	The statistical test(s) used AND whether they are one- or two-sided<br>*Only common tests should be described solely by name; describe more complex techniques in the Methods section.*
☐	☒	A description of all covariates tested
☒	☐	A description of any assumptions or corrections, such as tests of normality and adjustment for multiple comparisons
☐	☒	A full description of the statistical parameters including central tendency (e.g. means) or other basic estimates (e.g. regression coefficient) AND variation (e.g. standard deviation) or associated estimates of uncertainty (e.g. confidence intervals)
☐	☒	For null hypothesis testing, the test statistic (e.g. *F*, *t*, *r*) with confidence intervals, effect sizes, degrees of freedom and *P* value noted<br>*Give P values as exact values whenever suitable.*
☒	☐	For Bayesian analysis, information on the choice of priors and Markov chain Monte Carlo settings
☒	☐	For hierarchical and complex designs, identification of the appropriate level for tests and full reporting of outcomes
☒	☐	Estimates of effect sizes (e.g. Cohen's *d*, Pearson's *r*), indicating how they were calculated

Our web collection on statistics for biologists contains articles on many of the points above.

Software and code

Policy information about availability of computer code

Data collection	We did not use any code to collect data.
Data analysis	We used the following software:<br>ConsensusClusterPlus (v1.64.0)<br>Cyt interface in Matlab (Cyt3)<br>drc (v3.0-1)<br>Flowsom (v2.8.0)<br>Matlab<br>NONMEM (v7.4.4)<br>Phenograph algorithm (no version availble as this was implemented in the Cyt interface)<br>R (v3.6.1)<br>Standard BioTools CyTOF software v7.1

For manuscripts utilizing custom algorithms or software that are central to the research but not yet described in published literature, software must be made available to editors and reviewers. We strongly encourage code deposition in a community repository (e.g. GitHub). See the Nature Portfolio guidelines for submitting code & software for further information.

Data

Policy information about availability of data

All manuscripts must include a data availability statement. This statement should provide the following information, where applicable:

- Accession codes, unique identifiers, or web links for publicly available datasets
- A description of any restrictions on data availability
- For clinical datasets or third party data, please ensure that the statement adheres to our policy

Source Data are provided with this paper. Access to patient-level data is restricted due to data privacy laws but may be requested by completing a data access agreement, available on application to the data access committee (Data-access@bergenbio.com). Access will be granted exclusively to qualified investigators for appropriate non-commercial use that is expected to lead to a publication. Access will be subject to approval by a regional ethical committee to ensure that it is in line with lawful basis for processing, data protection regulations, and ethical standards. Access will be granted for purposes specified in the data access agreement for the time sufficient to fulfil those purposes. We aim to provide an initial response to data access requests within 3 weeks. Correspondence and materials requests should be addressed to S.L. (Sonja.Loges@medma.uni-heidelberg.de) and C.O. (cristina.oliva@bergenbio.com).

Research involving human participants, their data, or biological material

Policy information about studies with human participants or human data. See also policy information about sex, gender (identity/presentation), and sexual orientation and race, ethnicity and racism.

Reporting on sex and gender	We determined sex based on self reporting. There is no disaggregation of sex in our analysis due to the small sample size of the trial. At the time of the study set-up, no gender or sex-related analysis were planned. As this was primarily a study focussing on safety and tolerability of the study drug, no questionnaires in regards to sex and gender were implemented.
Reporting on race, ethnicity, or other socially relevant groupings	In addition to the lack of sex- and gender disaggregated data due to the study design, there are no data collected aiming to investigate correlations between e.g. response and race, ethnicity or other socially relevant groupings.
Population characteristics	Covariate-relevant characteristics:<br>6 treatment-naive patients and 30 relapsed/refractory patients (15 in 2L, 15 in >2L) were included for efficacy assessment.<br><br>Other population characteristics (which were not covariate-relevant) are listed in Table 1.
Recruitment	Patients were recruited at the discretion of the sites. We are not aware of any selection bias.
Ethics oversight	The study complied with all relevant legal and ethical regulations regarding the use of human study participants and was conducted in accordance with the Declaration of Helsinki and the International Council for Harmonisation (ICH) with Good Clinical Practice. Study centers and associated ethics committees/institutional review boards providing approval are listed below. The study was authorized by the Norwegian Medicines Agency (3 July 2014), the Federal Institute for Drugs and Medical Devices (Germany, 21 October 2014), the U.S. Food and Drug Administration (30 October 2014) and the Italian Medicines Agency (7 February 2018). All enrolled patients signed an informed consent form prior to study participation. Patients did not receive compensation.<br><br>Study Centers, Ethical approval<br>University of Iowa Hospitals and Clinics, Iowa City, USA: Human Subjects Office / Institutional Review Board, Hardin Library, Suite 105A,600 Newton Rd, Iowa City, IA 52242-1098, USA<br>The University of Texas M.D. Anderson Cancer Center, Houston, USA The University of Texas, MD Anderson Cancer Center, Institutional Review Board, 7007 Bertner Ave., Unit 1637, Houston, TX 77030, USA<br>University Hospital Frankfurt, Germany Ethik-Kommission des Fachbereichs Medizin der Goethe-Universität Frankfurt am Main, Theodor-Stern-Kai 7, 60590 Frankfurt am Main, Germany<br>Hannover Medical School, Germany Ethik-Kommission der Medizinischen Hochschule Hannover, Carl-Neuberg-Str. 1, 30625 Hannover, Germany<br>University Hospital Mannheim, Germany Ethik-Kommission II der Universität Heidelberg Medizinische Fakultät Mannheim, Haus 42, Ebene 3, Theodor-Kutzer-Ufer 1-3, 68167 Mannheim, Germany<br>University Hospital of Ulm, Germany Ethikkommission der Universität Ulm, Helmholtzstraße 20 (Oberer Eselsberg), D - 89081 Ulm, Germany<br>Azienda Sanitaria Ospedaliera Santa Croce e Carle, Cuneo, Italy Comitato Etico Interaziendale, Azienda Ospedaliera S. Croce e Carle, Via M. Zovetto, 18, 12100 Cuneo, Italy<br>San Martino Hospital, University of Genoa, Italy Comitato Etico Regione Liguria, IRCCS Policlinico San Martino, Sistema Sanitario Regione Liguria, Largo Rosanna Benzi, 10, 16132 Genova, Italy<br>Vito Facci Hospital, Lecce, Italy Comitato Etico ASL 3 Lecce, Via Miglietta, 5, 73100 Lecce, Italy<br>University Hospital Parma, Italy Comitato Etico dell'Area Vasta Emilia Nord (AVEN), Azienda Ospedaliero-Universitaria di Parma, Via Gramsci 14 , 43126 Parma, Italy<br>Haukeland University Hospital, Bergen, Norway. Regionale Komiteer for Medisinsk og Helsefaglig Forskningsetikk – REK Sør-Øst D, Gullhaugveien 1-3, 0484 Oslo, Norway

Note that full information on the approval of the study protocol must also be provided in the manuscript.

Field-specific reporting

Please select the one below that is the best fit for your research. If you are not sure, read the appropriate sections before making your selection.

[x] Life sciences [ ] Behavioural & social sciences [ ] Ecological, evolutionary & environmental sciences

For a reference copy of the document with all sections, see nature.com/documents/nr-reporting-summary-flat.pdf

Life sciences study design

All studies must disclose on these points even when the disclosure is negative.

Sample size

36 patients were included in the dose escalation cohort.
Justification for sample size in this cohort: The conventional 3+3 study design gives a 71% chance of escalation if the true but unknown rate of DLT is 20%, and <50% chance of escalation if the true but unknown rate of DLT is >30%.
36 patients were included in the bemcentinib+low dose cytarabine cohort (consisting of the study cohorts B2 and B5).
Justification for sample size in this cohort: Based on a one-sided, within-group test of proportions comparing an anticipated ORR of >20% against the null hypothesis rate of 5%, with power 80% and type I error 0.2 (suitable as evidence of a trend worthy of future study), a sample size of up to 14 evaluable patients was selected for B2. On the same basis, for B5 an initial sample size of 14 relapsed and 14 refractory AML patients was planned. However, upon regulatory review by the FDA, this was reduced to 20 R/R AML patients with 4 refractory AML patients. For this early-stage study, the efficacy analyses are secondary endpoints, and no account was taken of the multiplicity inherent in the assessment of several, presumably non-independent criteria, for ORR.

Data exclusions

We did not exclude any data.

Replication

Results need to be confirmed in a phase III trial.
It is not planned and not common to replicate phase II trials.

Randomization

This trial was not randomised because of its exploratory nature.
Covariate control is not necessary in an exploratory setting.

Blinding

We did not perform blinding because this is an open-label trial.

Reporting for specific materials, systems and methods

We require information from authors about some types of materials, experimental systems and methods used in many studies. Here, indicate whether each material, system or method listed is relevant to your study. If you are not sure if a list item applies to your research, read the appropriate section before selecting a response.

Materials & experimental systems

n/a	Involved in the study	
[ ]	[x]	Antibodies
[x]	[ ]	Eukaryotic cell lines
[x]	[ ]	Palaeontology and archaeology
[x]	[ ]	Animals and other organisms
[ ]	[x]	Clinical data
[x]	[ ]	Dual use research of concern
[x]	[ ]	Plants

Methods

n/a	Involved in the study	
[x]	[ ]	ChIP-seq
[x]	[ ]	Flow cytometry
[x]	[ ]	MRI-based neuroimaging

Antibodies

Antibodies used

The following antibodies were used, identified by their research resource identifiers (www.rrids.org), and detailed in Supplementary Figure 5:
AB_2938863, AB_2562837, AB_3661863, AB-2607573, AB_2921324, AB_2811085, AB_3661845, AB_2811089, AB_2861412, AB_2938861, AB_314494, AB_2847864, AB_2661790, AB_2562813, AB_3665424, AB_3661860, AB_2687654, AB_2893063, AB_2665397, AB_2687640, AB_2847866, AB_2811248, AB_2811100, AB_2744690, AB_2811246, AB_2847867, AB_390781, AB_2661832, AB_2661834, AB_2661826, AB_2938622, [2], AB_2062560, AB_11214201, AB_2847869, AB_2847863, AB_10989536.

Validation

Antibodies were validated by the manufacturer. Development, further validation and testing of the panel is described in Tislevol 2023, referenced in the paper.

Clinical data

Policy information about clinical studies
All manuscripts should comply with the ICMJEguidelines for publication of clinical research and a completedCONSORT checklist must be included with all submissions.

Clinical trial registration	EudraCT (2014-000165-46, 13 Jan 2014) and ClinicalTrials.gov (NCT02488408).
Study protocol	The full trial protocol is provided in Supplementary Note 1, with a version change history provided in Supplementary Table 2.
Data collection	Study centers: University of Iowa Hospitals and Clinics, Iowa City, USA, M.D.Anderson Cancer Center, Houston, USA, University of Frankfurt, Germany, University hospital Hamburg, Germany, University hospital Mannheim, Germany, University hospital Ulm, Germany, ASO. S. Croce e Carle, Cuneo, Italy, University of Genoa, Italy, Vito Facci Hospital, Lecce, Italy, University hospital Parma, Italy, Haukelands university hospital, Bergen, Norway<br><br>Patients were recruited between October 2014 and July 2021.<br><br>Data were collected until September 2022.
Outcomes	The primary objective was to assess the safety and tolerability of bemcentinib in combination with LDAC in AML patients unfit for intensive chemotherapy. We measured this outcome using standard clinical laboratory safety tests (hematology, biochemistry, coagulation, and urinalysis), physical examinations, vital signs (blood pressure, heart rate, respiration rate and temperature), and ECOG performance status were performed at baseline and throughout the study. Adverse events (AEs) were monitored for 28 days after the end of treatment and graded according to the National Cancer Institute Common Terminology Criteria for Adverse Events (NCI-CTCAE) version 4.0.<br><br>The secondary objective was to characterize the pharmacokinetic profile of bemcentinib and to explore the efficacy of bemcentinib in combination with LDAC. For pharmacokinetic analyses, we analysed particiants' blood and utilised liquid chromatography together with tandem mass spectrometry. We then computed AUC(0-T), C(av,ss), C(max,ss) and t(1/2). For efficacy assessment, we used objective response rate (ORR), disease control rate (DCR), objective response (OR), stable disease (SD) and unchanged disease status for 3 treatment cycles, relapse-free survival (RFS), event-free survival (EFS), and overall survival (OS).

Plants

Seed stocks	Not applicable
Novel plant genotypes	Not applicable
Authentication	Not applicable
