# Supplementary material for: Bemcentinib as monotherapy and in combination with low-dose cytarabine in acute myeloid leukemia patients unfit for intensive chemotherapy: a phase 1b/2a trial
Source: Nat Commun. 2025 Mar 23;16:2846. doi: 10.1038/s41467-025-58179-6 (PMC11930985; doi:10.1038/s41467-025-58179-6)
Supplement: Supplementary file 4 — Description of Additional Supplementary Files [file 41467_2025_58179_MOESM4_ESM.pdf]

Title: Supplementary Data 1

Description: Supplementary data tables for Dose Limiting Toxicities; Fatal, SAE, TEAEs related to bemcentinib; QTcF-related adverse events; TEAEs by system organ class; TEAEs leading to dose modifications.
